# Competitive resource allocation drives asynchronous and rapid nuclear multiplication in the malaria parasite

**DOI:** 10.1038/s41467-026-75378-x

**Published:** 2026-07-27

**Authors:** Patrick Binder, Aistė Kudulytė, Severina Klaus, Thomas Höfer, Ulrich S. Schwarz, Markus Ganter, Nils B. Becker

**Affiliations:** 1 Theoretical Systems Biology, German Cancer Research Center (DKFZ), Heidelberg, Germany; 2 Institute for Theoretical Physics, Heidelberg University, Heidelberg, Germany; 3 BioQuant, Heidelberg University, Heidelberg, Germany; 4 Center for Infectious Diseases—Parasitology, Medical Faculty, Heidelberg University, Heidelberg, Germany; 5 Present Address: Center for Infectious Diseases—Virology, Medical Faculty, Heidelberg University, Heidelberg, Germany

**Keywords:** Parasite development, Biological physics

## Abstract

The unicellular malaria parasite *Plasmodium falciparum* proliferates within red blood cells of its human host, where it generates approximately 20 new parasites within a two-day developmental cycle. Before cellularization and release of the daughter cells, the nuclei multiply in a shared cytoplasm. In stark contrast to highly synchronized nuclear division cycles seen in other developing eukaryotes, *Plasmodium* nuclear cycles desynchronize rapidly. Combining live-cell imaging with biophysical modeling, we elucidate the mechanism of desynchronization and study its impact on parasite proliferation. We find that standard models of autonomous nuclear cycles cannot account for the experimental data, and therefore desynchronization requires nuclear coupling. Competition for a limiting pool of proteins needed for DNA replication explains the data, provided that they are allocated sequentially to individual nuclei. Sequential allocation can be achieved by reversible but stable association of the resources with DNA. Remarkably, the resultant asynchronous nuclear cycles accelerate parasite proliferation by minimizing idling times of the resource. This mechanism may be a general strategy to maximize proliferation in suboptimal growth conditions. Together, our findings identify nuclear cycle asynchrony as a resource-efficient means to achieve rapid proliferation.

## Abstract (web summary)

Within red blood cells, *Plasmodium falciparum* forms cells with multiple nuclei that replicate asynchronously despite residing in a shared cytoplasm. In this study, the authors developed a biophysical model that explains how asynchrony is established.

## Introduction

**Fig. 1:**
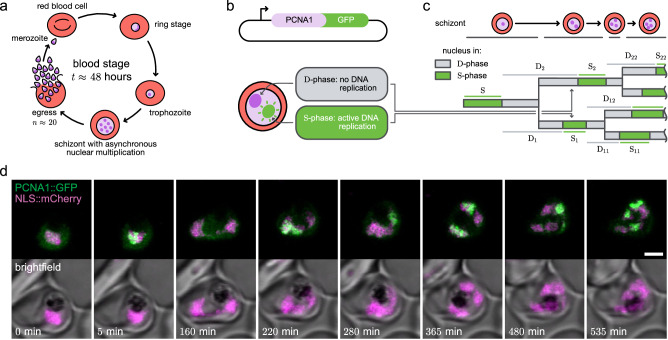
Blood-stage schizogony of the malaria-causing parasite *P. falciparum.* **a** Schematic of the asexual reproductive cycle. **b** Nuclear cycle sensor line, which episomally expresses a *P. falciparum* PCNA1::GFP fusion protein. PCNA1::GFP accumulates specifically in nuclei undergoing active DNA replication, enabling live-cell tracking of S-phase nuclei. **c** Lineage tree of tracked nuclei. Nuclear cycles are divided into S-phase (active DNA replication) and D-phase (interval between two subsequent S-phases). Phase subscripts 1, 2, 12, 22 denote descendant nuclei, e.g., 2 for the second daughter to enter S-phase and 21 for its first-entering daughter. **d** Time-lapse confocal image sequence of a single infected red blood cell. Asynchronous DNA replications are visible from 220 min onwards. NLS, nuclear localization signal. Scale bar, 2 μm.

Parasites, like other pathogens, are constantly attacked by the host immune system, and only a small inoculum is typically transmitted from one host to another. An effective survival strategy in this situation is rapid proliferation, in order to compensate for the losses and to recreate a population size that is sufficiently large to allow for successful transmission. This strategy is also employed by the unicellular eukaryotic parasites of the genus *Plasmodium*, including *P. falciparum*, which is responsible for the most virulent form of human malaria^1^. All clinical symptoms of malaria occur during the blood stage of the infection, where *P. falciparum* proliferates inside red blood cells (RBCs), causing a parasite burden which can exceed 10% of RBCs^2,3^. Proliferation within RBCs is achieved via the unique process of schizogony^4^. So-called merozoites invade RBCs and first develop into ring and trophozoite stages, during which the parasite grows and remodels the host RBC to evade host defenses. After approximately 30 h^5–7^, the parasite proceeds to the schizont stage, which is characterized by the multiplication of nuclei in a shared cytoplasm^4,8^. After 48 h, cellularization occurs and around 20 daughter merozoites are released (Fig. 1a).

In marked contrast to the pattern of proliferation seen in other multinucleated cells, such as the early *Drosophila melanogaster* embryo^9^ or the unicellular marine eukaryote *Sphaeroforma arctica*^10^, nuclear multiplication in *P. falciparum* does not follow a sequence of synchronous divisions with associated doublings in number. Instead, *P. falciparum* nuclei multiply asynchronously despite sharing a common cytoplasm^7,11–13^. Already at the stage of two nuclei, the time span between nuclear division and the subsequent DNA replication can vary considerably between sister nuclei^13^.

This asynchrony would not be possible if all nuclei were regulated by shared canonical eukaryotic cell cycle checkpoints^4,14^, and this has been taken as an indication that control of nuclear cycles in *P. falciparum* is local, not global^8^. Already in 1875, botanist E. Strasburger noted that individual nuclei in multinucleated cells organize their own “sphere of influence”^15^, hinting that cytoplasmic compartmentalization may be key for asynchrony. This notion is supported by data from the filamentous fungus *Ashbya gossypii*, where spatially separated nuclei show asynchrony^16,17^, but partially synchronize when brought into proximity^18–21^. By contrast, *P. falciparum* nuclei are always in very close proximity within RBCs, where any protein gradient equilibrates within milliseconds, precluding effective cytoplasmic compartmentalization. Alternatively, as homologs of the canonical G1-, S- and M-phase cyclins^22^, which would synchronize nuclei globally, appear to be absent in *Plasmodium*^23–25^, asynchrony may be due to purely nucleus-intrinsic mechanisms. These would give rise to nuclear cycles that are stochastic and independent, except for possible correlating effects of inheritance of nuclear components such as the centrosome^8^.

There is a rich literature on mathematical models that connect the heritable phenotypic plasticity of cells with their synchronization and population growth^26–30^. A recurrent theme is that the behavior of the cell population is determined by nutrient availability^31^, which affects the regulation of the cell cycle^32,33^ as well as the cellular growth speed^34,35^. Regarding *Plasmodium*, mathematical models have focused on disease progression within an entire host^36^ or human population^37^. However, it remains elusive how asynchronous nuclear cycles arise in and if they may be beneficial for the parasite. Here, we address these questions by combining quantitative analysis of high-resolution time-lapse microscopy data with a theory of the underlying biophysical mechanism. We focus on minimal models that incorporate the two experimentally resolved nuclear cycle phases^12^ as well as their coupling via inheritance and the sharing of cellular resources.

Strikingly, our data rule out models where individual nuclei cycle independently as well as models where nuclei are correlated solely via inheritance of nuclear factors. These findings imply that coexisting nuclei are affected by each other’s presence. Specifically, our data support a biophysical model where nuclei compete for a molecular resource in the form of a shared pool of one or several proteins involved in DNA replication. The regulation of nuclear multiplication is then achieved by a limiting pool mechanism, similar to mechanisms governing size regulation of cellular structures^38–41^. Thus, in contrast to previous hypotheses^8^, we provide evidence for global control of nuclear cycle regulation, which desynchronizes *P. falciparum* nuclei through competition for a limiting resource. We also find that nuclei that replicate their DNA sequentially use the resource more steadily, which can increase efficiency and speed up nuclear multiplication. Together, our results explain asynchrony of nuclear multiplication as a consequence of the need for economical and rapid proliferation to ensure life cycle progression of *P. falciparum*.

## Results

### Lineage tracking of nuclei in *P. falciparum*

To understand how asynchronous nuclear cycles arise, we first gathered quantitative experimental data on the timing of nuclear cycles. We relied on our *P. falciparum* reporter cell line, which can be used as a sensor for the nuclear cycle phases^13^. This line ectopically expresses the red-fluorescent protein mCherry fused to three nuclear localization signals, which effectively labels all nuclei in a schizont. In addition, the line ectopically expresses the DNA sliding clamp protein PCNA1, an essential component of the eukaryotic DNA replication fork ^42,43^, fused to green fluorescent protein (PCNA1::GFP). In *P. falciparum*, PCNA1::GFP accumulates transiently in individual nuclei, and we have previously shown that those nuclei undergo productive DNA replication during S-phase (Fig. 1b)^13^. Furthermore, blocking processive DNA replication leads to a prolonged nuclear accumulation of PCNA1::GFP^44,45^. This reporter line, thus, allows us to monitor DNA replication and nuclear division in individual nuclei of a schizont (Fig. 1c, d).

Using long-term time-lapse confocal microscopy, we previously tracked individual nuclear lineages, covering the initial S-phase as well as the division of the first two sister nuclei, in *N* = 55 cells (and one additional nuclear division in *N* = 25)^13^. To analyze these quantitative data, we divided the nuclear cycles phenomenologically into two consecutive phases (Fig. 1b, c): The (active) S-phase is defined by PCNA1::GFP accumulation in a given nucleus, and the remainder of a nuclear cycle is combined into a division phase (D-phase), during which no active DNA synthesis occurs. D-phase begins when the nuclear accumulation of PCNA1::GFP disappears, extends through nuclear division, and continues in each daughter nucleus until the respective ensuing S-phase begins. Thus, the S_*i*_-phase of a nucleus *i* is followed by two D-phases, D_*i*1_ and D_*i*2_, for its two daughter nuclei *i*1 and *i*2, respectively. The two D-phases share a time period from the end of DNA replication of the mother nucleus *i* to its division (Fig. 1c), which we did not resolve. We chose this two-phase description (similar to earlier work^12^), because it matches well with the resolution of our live-cell imaging data, where the two phases can be accurately quantified. By contrast, the G1, S, G2 and M phases of the canonical cell cycle are poorly defined in *P. falciparum*^4^ and appear ill-suited to describe asynchronous nuclear multiplication.

### Evidence for inter-nuclear coupling

Next, we used the observed nuclear cycle dynamics to ask if *P. falciparum* nuclei multiply autonomously, that is, without being affected by the presence of other nuclei or signaling in the cell. We started by considering a simple model (model 1) in which all nuclei are equally DNA-replication competent, and cycle regulation relies solely on non-inheritable, nucleus-intrinsic factors. Mathematically, these requirements lead to a two-phase branching process^46,47^, where each S- or D-phase is drawn independently from the corresponding empirical distribution. These distributions were derived from data of the first four experimentally observed cycle phases, collected from cells containing between one and four nuclei.

**Fig. 2:**
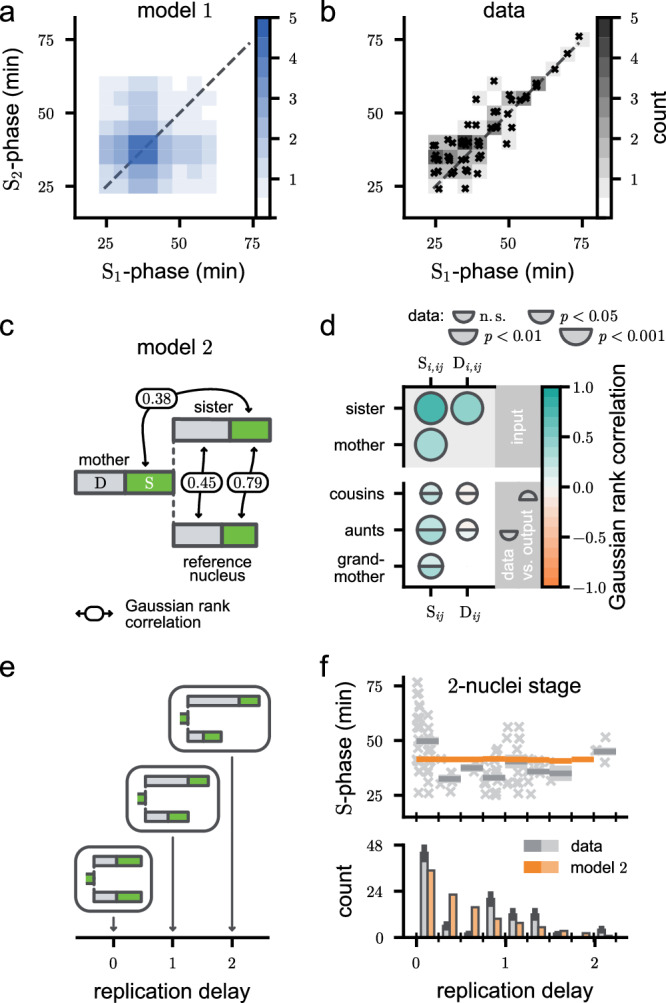
Predicted and experimentally observed nuclear cycle correlations in *P. falciparum.* **a** The two-phase branching process (model 1) fails to reproduce the positive correlations observed between sister S-phases at the 2-nuclei stage as shown in **b**. For (**b**), *r* = 0.87: Pearson correlation coefficient (*p* = 3.7 × 10^−18^, two-sided *t*-test), *N* = 55. **c** Schematic of model 2, which incorporates inheritance from mother to daughter nuclei, with included input Gaussian rank correlations. **d** Gaussian rank correlations, calculated as Pearson correlations between standardized phase durations of related cell pairs. Bottom-half disks: data (with *p*-value, two-sided *t*-test). Top-half disks: model 2. Gray background: input correlations. **e** Illustration of DNA replication delay between two sister nuclei, defined as $({\tau }_{{{{{\rm{D}}}}}_{2}}-{\tau }_{{{{{\rm{D}}}}}_{1}})/\langle {\tau }_{{{{{\rm{S}}}}}_{1}}\rangle$, where S_1_ is the sister S-phase that starts first. **f** S-phases vs. delay at the 2-nuclei stage. Gray crosses, lines and shading: data, mean, and its bootstrapped standard error, respectively; *N* = 102 total S-phases. Orange lines: model 2 prediction for the mean; *N*_sim_ = 10^5^ S-phases, standard error is smaller than line width. Bottom histogram: simulated and experimentally observed replication delays with bootstrapped standard error bars, obtained by binning the data shown at the top of the panel.

To test model 1, we measured the S-phase durations of sister nuclei at the 2-nuclei stage. Because nuclei are independent in model 1, it predicts no correlation between the S-phase durations (Fig. 2a). By contrast, the experimental data showed a positive correlation between sisters, especially when S-phases were longer (Fig. 2b). This provided evidence that nuclei in a *P. falciparum* schizont are not independent and their nuclear cycle is not intrinsically regulated, ruling out model 1.

The detected correlation between sister S-phases could be plausibly explained by the inheritance of factors from the mother nucleus. To investigate this possibility, we constructed model 2 as a Markovian bifurcating autoregressive (BAR) process^28–30,48^, in which the cycle phases of the sister nuclei depend on those of the mother nucleus, but are not directly affected by other nuclei in the cell. For instance, sister nuclei may inherit a bias for a longer S-phase from a mother with a long S-phase, and the opposite from a mother with short S-phase, which would correlate sister S-phases positively. Model 2 was parameterized via the Gaussian rank correlations between all mother and daughter nuclear cycle phases (see Sec. Methods, Supplementary Fig. 1 and Supplementary Note 2 for details). In our data, S-phases were not correlated with D-phases in the same or different nuclei (Supplementary Fig. 1c), while S-phases were positively correlated between mother and each daughter nucleus and between sisters.

By construction, model 2 reproduced the distributions of the two nuclear-cycle phases as well as the mother-daughter and sister-sister correlations present in the data (Fig. 2c). In addition, it successfully predicted the experimentally observed correlations between more distantly related nuclei. For instance, S-phases of aunts and nieces were correctly predicted to be positively correlated (Fig. 2d).

We then tested if model 2 reproduced other features of the experimental data, initially focusing on the prolonged S-phases of synchronously replicating sister nuclei^13^. To analyze pairs of sister nuclei, we considered the delay with which they enter S-phase. We defined the replication delay as the difference of the replication start times, normalized by the average duration of the first (leading) S-phase, i.e., $({\tau }_{{{{{\rm{D}}}}}_{2}}-{\tau }_{{{{{\rm{D}}}}}_{1}})/\langle {\tau }_{{{{{\rm{S}}}}}_{1}}\rangle$. Thus, a replication delay of 0 indicates synchronous entry into S-phase, a delay of 1 implies roughly sequential DNA replication, and a delay >1 indicates a gap between the end of S_1_ and the onset of S_2_ (Fig. 2e). Whereas our experimental data showed a 1.5-fold slowdown of S-phases in sister nuclei with a delay 0 (compared to positive delays), model 2 predicted a constant S-phase duration at all replication delays (orange lines) (Fig. 2f, top panel, gray points and lines).

To further test model 2, we compared the frequencies of the various replication delays (Fig. 2f, bottom panel). While model 2 predicted a gradual decrease of events with increasing replication delay, our experimental data showed a bimodal distribution with enriched replication delays of 0 and 1, and only few delays around 0.5. Because model 2 captured neither the prolonged S-phases of synchronous sister nuclei nor the bimodal distribution of delays, we ruled it out as an explanation for the observed behavior of nuclei in *P. falciparum*. We also excluded extended BAR models that incorporate more distant correlations, as these would not correspond to physiological mother–daughter inheritance.

Hence, our experimental data disqualify a large class of autonomous models in which the duration of the different phases of nuclear multiplication is determined independently or by inheritance of nucleus-intrinsic factors. These results strongly suggest that a physical mechanism couples the nuclei that coexist in the common cytoplasm.

### A limiting pool mechanism couples replicating nuclei

Next, we sought an explanation for how other nuclei in a given cell may affect the onset and timing of S-phase. A simple and parsimonious mechanism that would couple such DNA replicating nuclei is the competition for a shared resource needed for replication, *e.g*., one or several components of the DNA replication machinery.

Competition for a resource is central for so-called limiting pool mechanisms, which can regulate the size of cellular structures and organelles without the need for dedicated sensing pathways^38,39^: Their basic feature is that, as organelles grow, they incorporate building blocks from a cytosolic pool, thereby depleting it, and slowing their further growth. This mechanism has been shown to effectively regulate the size and relative scaling of cellular structures, including mitochondria, vacuoles, nuclei, centrosomes and flagella^41^, mitotic spindles^49^, as well as actin networks^40^.

Similar to the sharing of (or competition for) cellular building blocks, the nuclei in a *P. falciparum* schizont may share and compete for DNA replication cofactors^13^. This idea is supported by the observation that S-phases are prolonged in synchronously replicating nuclei. Also, classical fusion experiments with human cells exhibit a prolonged S-phase in the two nuclei, which suggests competition for a DNA replication resource^50^. Thus, in analogy to limiting pool mechanisms, we hypothesized that there exists ‘resource’ R, whose abundance is directly related to the speed of active DNA replication. Furthermore, we consider R to be free to diffuse and translocate between nuclei in the cell. The resource R is defined by these two properties, and it may correspond to one or multiple proteins of the DNA replication machinery, whose molecular identity we intentionally leave unspecified. Such a resource can potentially explain our observations: If R is distributed unevenly among the nuclei, a nucleus with little R would not replicate its DNA, which could cause the observed asynchrony. Alternatively, if R is distributed evenly but in insufficient amounts, synchronous S-phases would take longer.

Unlike the limiting pool mechanisms mentioned above, the resource R is not permanently incorporated into the nuclei but is released back into the cytoplasm after S-phase concludes, and can then be reused by other nuclei. Nevertheless, in a growing population of nuclei the initial pool of R would soon become a bottleneck for DNA replication, necessitating some form of resupply.

**Fig. 3:**
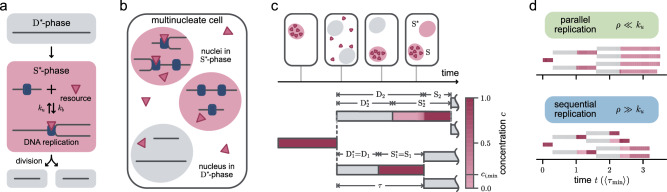
Biophysical model for competitive resource sharing (model 3). **a** In D^*^-phase, the replication machinery is unassembled. Upon entering S^*^-phase, the replication machinery is assembled, but active replication in addition requires loading of replication resource molecules. **b** S^*^-phase nuclei within a schizont compete for replication resource in the cytoplasm. **c** After the lagging daughter nucleus (subscript 2, top) enters S^*^-phase, it queues for resource to become available, remaining assigned to D-phase. It enters productive DNA replication (S-phase) after the leading daughter is done. **d** Example lineage trees. Parameters: *k*_*u*_ = 0.01 (sequential replication) and *k*_*u*_ = 100 (parallel replication); *r*(*t*) ≡ 1, $\langle {\tau }_{\min }\rangle=1$, *ρ* = 3.33, ${\sigma }_{{{{\rm{D}}^{*}}}}=0.02$, *k*_*b*_ = 10^6^.

In our previous study^13^, the slowdown of synchronous sister S-phases had led us to hypothesize that the nuclei may share some form of common good, but it remained unclear how this could be related to nuclear desynchronization. Thus, in the present work, we aimed to quantitatively explore the implications of a shared pool of a resource that limits DNA replication and whether this could explain how asynchronous nuclear cycles arise in *P. falciparum*. To this end, we constructed model 3, which couples nuclei via simple and biophysically plausible resource that is part of the DNA replication machinery at the replication fork (Fig. 3a). Model 3 posits that nuclei exist in the DNA replication-competent S^*^-phase, or in D^*^-phase. (These differ somewhat from the experimentally observed phases S and D, as explained below.) Unbound replication resource R translocates freely between the nuclei and the cytoplasm, equilibrating rapidly within the entire cell (Fig. 3b). In any S^*^-phase nucleus *i*, R can reversibly associate with the DNA replication forks F_*i*_ with binding and unbinding rates *k*_*b*_ and *k*_*u*_, respectively, forming the active replication fork complex RF_*i*_:

1 $${{{\rm{R}}}}+{{{{\rm{F}}}}}_{i} \mathop{\rightleftharpoons}_{{k}_{u}}^{{{k}_{b}}}{{{{\rm{R}}}}{{{\rm{F}}}}}_{i}.$$

This scenario is feasible, as we have shown that, e.g., PCNA1::GFP transiently accumulates in those nuclei that replicate their DNA^13^. It may appear that the first-order binding of Eq. (1) would be appropriate only if R were a single protein. However, it was demonstrated in ref. ^51^ that the collective activation of the multicomponent DNA repair machinery is effectively first-order, even though the binding of individual components is highly cooperative, at much higher rates. Thus Eq. (1) describes the binding of one or multiple factors that together enable active DNA replication.

In turn, active forks RF_*i*_ replicate DNA, such that the DNA amount *g*_*i*_ in a nucleus *i* increases from 1 to 2 haploid genomes during each S^*^-phase. This occurs via transient sequestration, but without consumption of R. The DNA synthesis speed ${\dot{g}}_{i}$ is proportional to the active fraction *c*_*i*_ of forks, which we write as ${\dot{g}}_{i}=\rho c_i$ with *ρ* denoting the DNA replication speed at full activation. S^*^-phase ends when a nucleus has fully replicated its DNA. When the resource is abundant this takes a minimum time 1/*ρ*.

After S^*^-phase, the replication forks disassemble, and resource R is instantaneously and irreversibly released. Then, two stochastic D^*^-phases ensue, spanning nuclear division and ending at the start of S^*^-phase in each of the daughter nuclei, respectively. As schizogony proceeds, nuclear cycles successively desynchronize in model 3, due to both the randomness of D^*^-phase durations ${\tau }_{{{{\rm{D}}^{*}}}}$ (standard deviation ${\sigma }_{{{{\rm{D}}^{*}}}}$), and variability in DNA replication speed of different nuclei resulting from varying available resource. On average, a full nuclear cycle lasts $\langle {\tau }_{\min }\rangle=1/\rho+\langle {\tau }_{{{{\rm{D}}^{*}}}}\rangle$ when resource is abundant. For a full account of model 3, including defining equations, see Methods and Table 1.

To relate this model to experimental data, we used PCNA1::GFP intensity^13,45^ as a proxy for active DNA replication and thus, for binding of resource R. The model accounts for the fact that active DNA replication is detectable only above a certain threshold of PCNA1::GFP intensity, by assigning a nucleus *i* to S-phase when activated forks exceeded a detection threshold ${{c}}_{i} > c_{i,\min}$. All other nuclei are assigned to D-phase. Thus, each S^*^-phase contains a detected S-phase (with productive DNA replication), while each D^*^-phase is contained within a detected D-phase (Fig. 3c). Notably, an S-phase can begin well after the enclosing S^*^-phase if resource is not immediately available in sufficient amounts.

### Resource binding kinetics and abundance control the synchrony of nuclear multiplication

Next, we investigated how synchronous or asynchronous nuclear cycles arise in model 3. We focus on a regime characterized by a high affinity of resource for the DNA replication fork, *k*_*u*_/*k*_*b*_ ≪ 1, and rapid binding, *k*_*b*_/*ρ* ≫ 1. As it turns out, in this regime the amount of available resource *r* relative to the number of replicating nuclei ${n}_{{{{\rm{S}}^{*}}}}$ in a cell, determines the dynamics of nuclear cycling. In particular, we call a cell resource-limited whenever the number of replicating nuclei is larger than the available resource ${n}_{{{{\rm{S}}^{*}}}} > r$ (in our rescaled units, see Methods). In such cells, although essentially all available resource is bound to replication forks (see Supplementary Note 3), one or more of the S^*^-phase nuclei must replicate at a reduced rate ${\dot{g}}_{i} < \rho$ for lack of resource.

Now consider a nucleus *i* in a resource-limited cell as it enters S^*^-phase. R is already fully in use by other actively running S-phases, and nucleus *i* can begin its DNA replication when R from other nuclei becomes available. How quickly this happens depends on the kinetics of R release. Fast release, *k*_*u*_ ≫ *ρ*, equilibrates R quickly among all nuclei, enabling immediate DNA replication in nucleus *i* while slowing other S-phases. Thus, fast resource release leads to parallel DNA replication in S^*^-phase nuclei. Conversely, slow release, *k*_*u*_ ≪ *ρ*, makes already-DNA replicating nuclei retain all bound resource, and S-phase in nucleus *i* is delayed until resource R is freed at the end of some other S^*^-phase. Hence, slow release enables sequential DNA replications. Between these extremes, a regime of intermediate resource release kinetics exists, where nuclei can start DNA replication after a moderate delay.

In sum, as the unbinding rate *k*_*u*_ decreases, model 3 interpolates between parallel and sequential modes of DNA replication (Fig. 3d). A shortage of resources results in prolonged, synchronous S-phases for high *k*_*u*_ (parallel mode), but may yield asynchronous S-phases for low *k*_*u*_ (sequential mode). Notably, resource abundance negates this effect, as all nuclei can immediately begin DNA replication in either mode of DNA replication, hiding the resource release kinetics. Thus, while parallel and sequential regimes of model 3 depend only on the kinetics of resource release, the actual occurrence of synchronous and asynchronous S-phases in a population of nuclei also depends on the amount of available resource.

### Nuclear multiplication in exponentially growing populations

*P. falciparum* exhibits exponential nuclear multiplication and DNA synthesis over several generations of nuclei (Supplementary Fig. 2,^6,13,52^). Before challenging model 3 with experimental data, we first consider the artificial but tractable setting of sustained exponential nuclear multiplication. Sustained exponential multiplication requires that resource *r* = *r*(*t*) also increase exponentially, as the overall DNA synthesis rate in the cell is at most *r*(*t*)*ρ* (cf. Methods, Eq. (8)). We incorporate this requirement by setting

2 $$r(t)=\zeta g(t),$$

where the constant resource availability parameter *ζ* quantifies how well the synthesis of resource can keep up with the increasing total amount of DNA *g* = ∑_*i*_*g*_*i*_.

As a proxy for nuclear multiplication, it is useful to consider the total rate of DNA synthesis $\dot{g}=\rho {c}$, where *c* = ∑_*i*_*c*_*i*_ (cf. Eq. (8) in Methods). To relate DNA synthesis to the abundance of resource, we introduce the resource utilization parameter

3 $$\eta \equiv {c}/r$$

as the instantaneous fraction of available resource used for DNA replication. (Intuitively, *η* = 1 corresponds to optimal resource usage, while *η* < 1 indicates unused potential for DNA replication.) Combining with Eq. (2) we finally obtain

4 $$\dot{g}=\rho \eta r=\lambda g,$$

where

5 $$\lambda \equiv \rho \eta \zeta$$

is the instantaneous exponential DNA replication rate, expressed in terms of the intrinsic synthesis rate *ρ*, external control by resource availability *ζ*, and resource utilization *η* resulting from the timing of nuclear cycle phases. Importantly, because DNA and nuclei increase in parallel, *λ* is also the (smoothed) nuclear exponential growth rate.

**Fig. 4:**
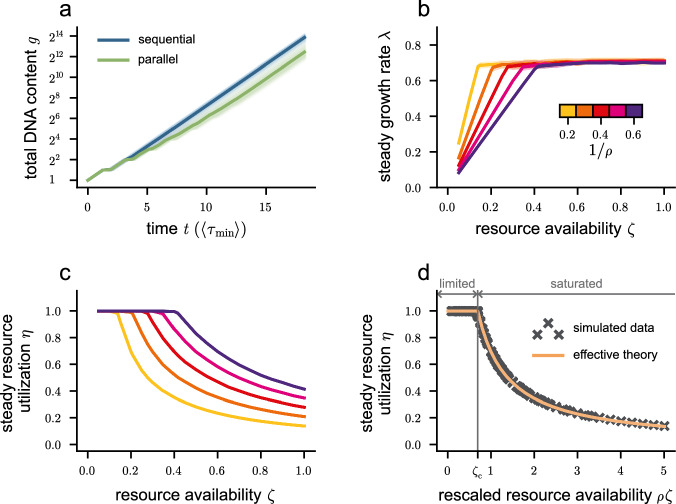
Asymptotic regime of nuclear multiplication in model 3. **a** Time course of total DNA content *g*(*t*), showing that convergence towards steady exponential growth is slower in parallel mode than in sequential mode. Lines, shading: simulation mean and overlaid realizations, showing the full accessible range. **b** Steady growth rates *λ* vs. resource availability *ζ*. Growth rates saturate to *λ*_max_ at the critical *ζ*_*c*_ = *λ*_max_/*ρ*. Colors: inverse replication rates corresponding to the non-limited S^*^-phase fractions. Shading: standard errors. **c** Steady resource utilization *η* vs. *ζ*. *η* decreases proportionally to 1/*ζ* above *ζ*_*c*_. Colors and shading as in (**b**). In (**b**) and (**c**), sequential and parallel mode curves are identical. **d** Collapse of simulation results onto the theoretical prediction given by Eq. (5) (yellow line). Crosses represent a simulation with a specific set of parameters (*ρ*, *ζ*, *k*_*u*_). For (**a**–**d**), *N*_sim_ = 480 lineage trees containing approximately 3 ⋅ 10^4^ S^*^-phases were generated per parameter set. The growth rate *λ* was determined by fitting an exponential to *g*(*t*) over the last 3 generations; *η* was obtained as *c*/*r* at the end of the simulation. Parameters: $\langle {\tau }_{\min }\rangle \equiv 1$, ${\sigma }_{{{{\rm{D}}^{*}}}}=0.1$, *k*_*b*_ = 10^6^, *k*_*u*_ = 0.01 (sequential mode) and *k*_*u*_ = 100 (parallel mode). In (**a**), *ζ* = 0.17 < *ζ*_*c*_ and *ρ* = 3.33.

We next explore the implications of these results. As time progresses, randomness in the D^*^-phase durations and S-phase progress gradually desynchronize nuclear cycles and, eventually, Eq. (4) attains a state of steady growth, which is characterized by exponentially increasing DNA content, total resource, and nuclear number, but constant fractions of the cycle phases within the population (Fig. 4a). We have determined the steady-growth values of *η* and *λ* depending on the resource (Methods). As the resource availability *ζ* is increased from 0, the steady growth rate first increases linearly, while the resource utilization initially remains at *η* = 1. At a critical value *ζ*_*c*_, the growth rate then saturates to a maximum value ${\lambda }_{\max }$ (Fig. 4b, Supplementary Note 4). With even more resource, the utilization then decreases as *η* ∝ 1/*ζ* (Fig. 4c, see also Methods).

The intrinsic proportions of the nuclear cycle phases affect the long-term growth rate (Fig. 4b, c). This is because shortening the S^*^-phase reduces the fraction of nuclei that require resource at any given time, effectively increasing availability of the resource. Interestingly, the resource release kinetics *k*_*u*_ have no bearing on the eventual steady growth rate (see Methods, Eq. (11)); any initial difference in growth rate between parallel and sequential modes of DNA replication disappears once nuclei are fully desynchronized. This is seen in Fig. 4b, c, where sequential and parallel mode simulations give identical results.

To test our effective theory for steady growth, we combined simulation data from a wide range of the parameters *ρ*, ${\sigma }_{{{{\rm{D}}^{*}}}}$ and *ζ*, spanning resource limited and non-limited cells, at slow, intermediate or fast release kinetics, in Fig. 4d. The data collapse onto a master curve given by Eq. (5), confirming the central tenet that nuclear cycles fully desynchronize in the long-time limit. For details on the simulation procedure, see Supplementary Note 6.

**Fig. 5:**
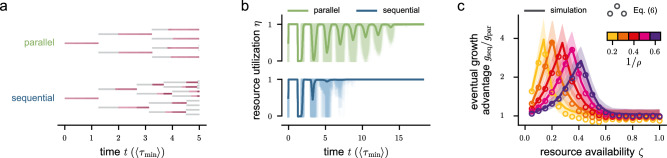
Initial phase of nuclear multiplication in model 3, highlighting the impact of replication mode on resource utilization and growth. **a** Example lineage trees for parallel and sequential replication modes. **b** Time course of resource utilization *η*. Transient drops in *η* (gaps) due to synchronized D^*^-phases persist longer in parallel mode. Lines, shading: simulation mean and *N*_sim_ = 480 single realizations, showing the full range. **c** The eventual growth advantage of sequential mode is maximized at intermediate resource availability. Lines, shading: simulation mean and standard errors of *g*_seq_/*g*_par_ at the end of the simulation, respectively, *N*_sim_ = 480. Circles: prediction by Eq. (6). Parameters as in Fig. 4, and in (**a**, **b**) as in Fig. 4a.

Having assessed the behavior of model 3 in long-term steady growth, we next investigate what shapes the initial growth phase, moving closer to the biologically relevant setting. Nuclear multiplication begins with a single (trivially synchronized) nucleus, and then desynchronizes at a rate depending on the resource release rate *k*_*u*_ (Fig. 5a). The crucial feature that distinguishes partially synchronized from fully asynchronous populations is the existence of time periods where most of the nuclei are in D^*^-phase, not binding resource *r*, which are visible as an intermittent drop (gap) in resource utilization *η*. Such gaps are more prominent and persistent in populations of nuclei that replicate DNA in parallel mode, and where cycles remain synchronized for much longer (Fig. 5a, b). During gaps, DNA replication $\dot{g}$ also drops (cf. Eq. (4)), which appears inefficient. To quantify the impact of gaps on the speed of nuclear multiplication, we obtained from Eq. (4) the ratio between total DNA content from sequential vs. parallel replication mode

6 $$\frac{{g}_{{{{\rm{seq}}}}}(t)}{{g}_{{{{\rm{par}}}}}(t)}=\exp \left\{\rho \zeta \int_{0}^{t}[{\eta }_{{{{\rm{seq}}}}}({t}^{{\prime} })-{\eta }_{{{{\rm{par}}}}}({t}^{{\prime} })]d{t}^{{\prime} }\right\},$$

which shows that it is the time integrated resource utilization that determines the overall speed advantage one replication mode may possess over the other.

In simulated populations, we found that the sequential DNA replication mode has a net utilization advantage *η*_seq_ − *η*_par_ ≥ 0 during the first few nuclear cycles. Importantly, as shown in Fig. 4a and predicted by Eq. (6), the transient utilization advantage leads to a persistent growth advantage (Fig. 5c). The growth advantage depends on the resource availability: In the resource saturated regime, *ζ* ≫ *ζ*_*c*_, nuclei replicate at maximal rate during the entire transient desynchronization period regardless of resource release kinetics, leaving no advantage. At very low resource availability *ζ* ≪ *ζ*_*c*_, the advantage of sequential replication also disappears, as in that limit, *λ* ∝ *ζ* (Eq. (5)). At intermediate availability, the sequential mode has a sizable advantage, which can reach threefold for plausible values *ρ* ≃ 2, *ζ* ≃ 0.3, and ${\sigma }_{{{{\rm{D}}^{*}}}}\simeq 0.1$ (Fig. 5c), and may be even larger if desynchronization is slowed further by lower D^*^-phase variability.

Between the extremes of sequential and parallel DNA replication lies a regime of intermediate resource release kinetics *k*_*u*_ ≃ *ρ*, which leads to an intermediate prevalence of DNA replication gaps and desynchronizes nuclei at intermediate speed (Supplementary Fig. 3a, b). The growth advantage compared to the parallel mode remains constant from the sequential regime up to *k*_*u*_ ≃ *ρ* and then decreases (Supplementary Fig. 3c).

Taken together, in model 3, the sequential mode of DNA replication enables faster nuclear multiplication when resources are not abundant. It does so by queuing replication-competent S^*^-phase nuclei for DNA replication during the first few generations. The queuing delays the onset of DNA replication in some nuclei, but it speeds DNA replication in already replicating nuclei, keeping the available resource in full use. The released resource is highly likely to be reused immediately in a queued nucleus, thereby avoiding gaps. Thus, queuing leads to more efficient resource usage and ultimately, to a persistent growth advantage.

### A limiting pool mechanism can explain asynchronous nuclear multiplication in *P. falciparum*

Sequential DNA replication is a strategy to accelerate the initial rounds of nuclear replication, in a scenario when saturating amounts of the resource R would not be economical or when they are not yet produced. Also, our tracking data show that nuclei in *P. falciparum* desynchronize early, hinting that they may be using this strategy. To test whether a limiting pool mechanism can indeed explain desynchronization and provide speed benefits in *P. falciparum*, we aimed to quantitatively reproduce our experimental data using simulations of model 3. In particular, the model simulations should exhibit the key features of prolonged S-phases of synchronous sister nuclei as well as a bimodal distribution of replication delays.

Initial exploration showed that the simple exponential resource model Eq. (2) supplies too much available resource towards the end of schizogony to match the data. This is consistent with our previous experimental finding that although nuclei initially multiply exponentially (Supplementary Fig. 2), the multiplication rate slows down towards the end of schizogony^13^. We hypothesized that the speed decrease is due to a decreasing availability of resource R to individual nuclei. Indeed, our time-lapse live-cell imaging data showed saturating levels of PCNA1::GFP (controlled by the PfCRT promoter) and 3xNLS::mCherry (PfHSP86 promoter) over time^13^. We used these proteins as a proxy for the resource abundance in model 3, by fitting a sigmoidal increasing function *r*(*t*) to the time courses and replacing Eq. (2) with it (Methods and Supplementary Fig. 4a, b). The fitted time course fixes the timing of saturation, while the long-term limiting resource amount *ξ* is allowed to vary between parasites (Supplementary Fig. 4c).

We also found the overall durations of nuclear multiplication to vary considerably between parasite cells^7,13,52^. This variation could not be reproduced by stochasticity coming solely from the D-phases in the model. We ascribed it to cell-to-cell differences in resource abundance, which we incorporate in the model by sampling *ξ* per cell from a uniform distribution with adjustable range (*ξ*_low_, *ξ*_high_).

The so-extended model 3 was then fitted to the experimental joint distribution of the phases S_*i*_, D_*ij*_ and the sister delay between S_1_ and S_2_, using Approximate Bayesian Computation (ABC). There were a total of seven adjustable model parameters, covering binding kinetics *k*_*u*_, *k*_*b*_, DNA replication speed *ρ*, D-phase variability ${\sigma }_{{{{\rm{D}}^{*}}}}$, resource availability range *ξ*_low_, *ξ*_high_, and an overall nuclear cycle time scale.

All parameters were identifiable in ABC, concentrating around unique maximum a-posteriori (MAP) values, with the notable exception of *k*_*u*_, which was bounded from above only (Supplementary Fig. 5). The binding rate *k*_*b*_ was inferred to be fast but finite, and resource variability was pronounced. Details of the model extensions and the Bayesian fit procedure are given in the Methods.

**Fig. 6:**
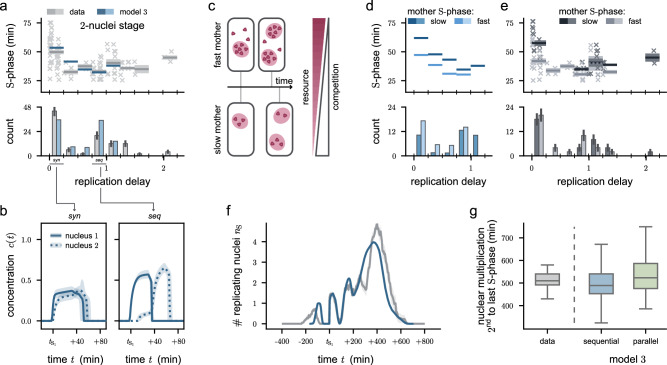
Model 3 captures nuclear multiplication dynamics in *P. falciparum* schizonts. **a** S-phase duration versus replication delay. Gray: replotted data and error bars from Fig. 2f. Blue: best-fit model 3 in sequential mode, reproducing the prolonged simultaneous S-phases and the depletion of intermediate delays; *N*_sim_ = 10^5^ sister pairs, standard errors are smaller than line width. **b** Fraction of active resource-fork complexes c at the 2-nuclei stage. Synchronous sisters (left) share resource and replicate slowly; delayed sisters (right) queue for resource and then replicate quickly. Lines and shading: simulation median and interquartile range, respectively. **c** Stratification according to mother S-phase duration. Fast (slow) mothers are predicted for high (low) resource abundance. **d** Stratified daughter S-phases statistics in model 3. Dark/light: slow/fast mother S-phase, respectively. **e** As (**d**), but using the experimental data shown in (**a**). Fast mothers are defined by S-phase $\le 50\,\min$, yielding *N*_fast_ = 42 and *N*_slow_ = 36. Means and histograms are shown with bootstrapped standard error bars. **f** Temporal profiles of the number of replicating nuclei. Desynchronization is similar in data and model 3. Colors as in (**a**). Profiles are aligned to the onset of the first S-phase in cells with two nuclei ${t}_{{{{{\rm{S}}}}}_{1}}$. Shading shows bootstrapped standard errors of the mean. **g** Time to complete nuclear multiplication, from start of S_1_ to end of last S-Phase. Box plots: center line indicates the median, box limits represent the upper and lower quartiles, and whiskers extend to the farthest data points within 1.5 times the interquartile range from the box limits. In (**a**, **b**, **d**, **f**, **g**), 10^5^ realizations were simulated, stopping at 24 nuclei. $\langle {\tau }_{\min }\rangle=88\,\min$. In unscaled units, $\rho=0.05\,{\min }^{-1}$, ${\sigma }_{{{{\rm{D}}^{*}}}}=8\,\min$, *ξ* ~ Uniform (0.8, 1.5), *c*_*i*,min_ = 0.15. $({k}_{u},{k}_{b})=(4.8\cdot 1{0}^{-5},0.23)\,{\min }^{-1}$ and $(1.1,5500)\,{\min }^{-1}$ for best-fit (sequential mode) and parallel mode, respectively.

When we tested the best-fit extended model 3, we found that it successfully reproduced both the prolonged S-phases of synchronously DNA replicating nuclei, and the bimodal distribution of replication delays at the 2-nuclei stage (Fig. 6a). Critically, the inferred upper bound for the unbinding rate implies that our experimental data were compatible only with slow resource release (*k*_*u*_ ≪ *ρ*), i.e., with model 3 in sequential replication mode but not in parallel mode (Supplementary Fig. 5).

This suggests that *P. falciparum* cells contain a limiting amount of a resource needed for DNA replication and that this resource can be stably sequestered within a nucleus. In this scenario, a lagging sister nucleus will be queued for replication until the leading sister finishes S^*^-phase and releases sufficient amounts of resources. These sister pairs show a replication delay of 1, together contributing the upper mode of the bimodal S-phase distribution (cf. Fig. 6a). In some cases, both sisters enter S^*^-phase with a delay shorter than the (fast but finite) resource binding timescale ${({k}_{b}r)}^{-1}$, which leads to synchronous S-phases, because the lagging sister starts before the leading sister has had time to sequester all resource. These sisters show short replication delays ≃0, contributing the lower mode of the distribution. If one sister nucleus enters S^*^-phase just a little later, it will be queued for the full leading S^*^-phase, which leads to the lack of delay values between 0 and 1. Moreover, only the synchronous S-phases distribute the limited resource between sisters, incompletely activating their respective replication forks. Therefore, only the synchronous S-phases are slowed significantly (Fig. 6b).

To put model 3 to a more stringent test, we challenged it to predict features of the data not used for calibration. First, although model 3 is calibrated purely on single-nucleus distributions (Supplementary Fig. 4d, e) and correlations between sisters (Fig. 2b), it successfully reproduced phase correlations with other related nuclei, including mothers and cousins (Supplementary Fig. 4f).

Next, to further probe the resource competition mechanism, we stratified mother–daughters lineages into two equal subsets according to the replication speed of mother nuclei (Fig. 6c). The model predicts that fast mother nuclei occur predominantly in cells with high resource availability, so that the synchronously replicating daughter nuclei in these cells slow down only slightly. Conversely, slow mother S-phases are expected to occur in resource-poor cells, in which synchronous daughter S-phases slow down severely, while sequential daughter S-phases should remain relatively fast (as they can use the available resource exclusively). Simulated data from the best-fit extended model 3 agreed with these expectations (Fig. 6d). Strikingly, the stratified daughter S-phases of our experimental dataset also fully fit to this prediction (Fig. 6e): Synchronous daughters of slow (but not fast) mothers exhibited a severe slowdown of DNA replication. We also found that the few sister nuclei with a replication delay of around 0.5 all came from a fast mother. These results further support the hypothesis that competition among nuclei for a limiting resource drives S-phase timing.

Finally, to test model 3 beyond the 2-nuclei stage, we assessed whether it can capture the rapid desynchronization of *P. falciparum* nuclei as multiplication continues. Using high-resolution time-lapse microscopy, we recorded DNA replication activity in *P. falciparum* schizonts for the full duration of nuclear multiplication. Figure 6f shows the number of concurrent S-phases vs. time, averaged over *N* = 26 parasites (Fig. 6f, gray line). The peak of  ≈ 1.4 simultaneous S-phases at the second S-phase ${t}_{{{{{\rm{S}}}}}_{1}}$ shows that a subset of DNA replications at the 2-nuclei stage were synchronous, consistent with the partial synchrony observed in the tracking data (Figs. 2d and 6a). A DNA replication gap with most nuclei in D-phase is observed after the 2-nuclei stage (*t* ≃ 100min), but no further distinct gaps appear after the 4-nuclei stage (*t* ≃ 200min), demonstrating rapid desynchronization.

To generate the corresponding profiles for model 3, we specified a termination condition for the simulated nuclear lineage trees. Because we had previously found that one round of *P. falciparum* schizogony produces *n* ≃ 24 daughter nuclei^13^, we opted to prohibit further entries into S^*^-phase after 23 S^*^-phases have been initiated. Effectively, this implements a counter mechanism, which we had found previously to be most compatible with experimental correlations between duration and progeny number in *P. falciparum* schizogony^13,52^.

Simulating model 3 to termination with MAP parameters, we found that a fraction of nuclei are queued for replication in each generation. This queuing successfully reproduced the rapid desynchronization from the 2-nuclei stage onward, including the loss of the gaps after four nuclei, and the overall time needed for completing nuclear multiplication (Fig. 6f). We then also simulated parallel replication by using a parameter set derived from the MAP parameters by increasing *k*_*u*_ while fixing the dissociation constant *k*_*u*_/*k*_*b*_ and all other parameters. Similar to model 2, in model 3 with the parallel-mode parameters, gaps with a majority of nuclei in D-phase extend over several nuclear cycles, showing much slower desynchronization than observed experimentally (Supplementary Fig. 6).

When we conducted an independent set of experiments using a different microscope offering higher resolution (Zeiss LSM900 equipped with an Airyscan 2 detector, see Methods), we observed that nuclear cycles were consistently slower by about 33% (Supplementary Fig. 7a). This overall reduction in speed is likely due to increased phototoxic effects compared to the spinning disk PerkinElmer UltraVIEW VoX microscope used for the initial set of experiments. More interestingly, the new dataset showed alterations in nuclear cycle timing compared to the initial dataset. The slow-down of synchronous sister S-phases as well as the bimodal distribution of replication delays was reproduced, but fewer events with a delay around 1 were detected (Supplementary Fig. 7b). Also, nuclear multiplication was considerably more synchronous for the entire duration of schizogony (Supplementary Fig. 7c). Remarkably, these divergent observations are compatible with model 3, under conditions where the nuclear phases are generally slowed down (in this case by phototoxicity), but production of resource is unchanged. More precisely, when lengthening average S^*^- and D^*^-phases by 33% while keeping the time course *r*(*t*) and all other best-fit parameters unchanged, model 3 successfully reproduces the 2-nuclei stage statistics, the correlation pattern between the cycle phases, and the slowed desynchronization of the repeat experiment (Supplementary Fig. 7b–d). The increased synchrony in this shifted parameter regime arises in the model as follows. When nuclei replicate slower, effectively resource abundance is increased, and competition for resource becomes less severe. With more abundant resource, the lagging sister at the 2-nuclei stage can then begin S-phase sooner using newly produced resource, which reduces the delay. Furthermore, nuclei at later stages desynchronize more slowly due to generally reduced queuing. Thus, by impeding nuclear cycle progression in ways unrelated to DNA replication speed, the resource R in model 3 becomes less limiting, which reproduces all changes observed in our second dataset.

Taken together, our data support the following interpretation: When *P. falciparum* enters the schizont stage, one or more components of the DNA replication machinery (resource) are limiting for replication. This resource remains limiting for the duration of nuclear multiplication despite its continued production. Resource limitation enqueues nuclei for entering S-phase, which rapidly desynchronizes the nuclear cycle phases. The resulting asynchronous DNA replication ensures optimal utilization of the replication machinery, which provides an overall speed benefit. When nuclear multiplication is slowed down independently, the shared resource may no longer be limiting, leading to much slower desynchronization of the nuclear cycles. This does not constitute a decrease in efficiency, since under the non-limited conditions, desynchronization would not yield a speed benefit.

## Discussion

In this study, we have addressed the open question of how asynchronous nuclear multiplication arises in *P. falciparum* schizonts, and what benefits it may have. After comparing statistical and biophysical models with imaging data, our work suggests that *P. falciparum* has evolved resource sharing between nuclei in a common cytoplasm as a way to achieve efficient proliferation. The experimentally supported model features a limiting pool of protein resources involved in DNA replication, for which nuclei compete through reversible association with DNA. The model successfully reproduces the slowdown of synchronous S-phases, the lack of partly overlapping S-phases, and the rapid desynchronization of nuclear cycles observed in *P. falciparum*^7,13^. This requires that protein resources are sequestered stably in nuclei during active DNA replication, so that other S^*^-phase nuclei are queued for replication. A central finding is that the sequential mode of DNA replication can speed up nuclear multiplication by decreasing the time the resource is not in use. Our results suggest that asynchronous multiplication of nuclei in *P. falciparum* is not a sign of autonomy, as in fungal hyphae^53^, and they argue against an entirely local control of *Plasmodium* nuclear cycles^8^. Rather, asynchrony appears as a manifestation of a physical coupling of nuclei via competition for the replication resource.

While the molecular identity of resource R in the model is left unspecified, our available data are consistent with R consisting of cofactors that are immediately involved in DNA replication and translocate between nuclei. While PCNA1 appears to fit these criteria (cf. Fig. 1d), it remains to be tested whether PCNA1 can become limiting. PCNA1 is one of multiple components of the replication machinery, which collectively control the rate of DNA replication. This has the likely consequence that PCNA1 abundance would have to be drastically reduced to affect the DNA replication, as found for DNA repair^51^. It could well be that no single component of the replication machinery is in fact the limiting resource. Identifying the minimal set of essential components of R is an interesting avenue for future work. For instance, the origins of replication within a given nucleus have been shown to compete for multiple proteins involved in origin firing, e.g., Sld2, Sld3, and Sld7, which leads to some origins being activated early during S-phase and some origins that fire later^54,55^. These proteins are thus further potential candidate components of R.

Apart from competition for the replication resource R, other mechanisms could plausibly couple nuclei. First, coupling could arise through a pool of a nutrient-like resource H, which is initially full and then successively depleted as nuclei multiply (unlike R in our model). In this scenario, the nutrient amount sets the final size of the system, very similar to limiting pool mechanisms for organelle growth^38^. However, H limitation would set in only after a certain number of nuclei have been produced, failing to generate the correlations between early nuclei seen in Fig. 2b. Second, DNA replication also depends on various consumable but renewable resources N, such as histone proteins and nucleotides. If running S^*^-phases exhausts a putative N pool, then N resupply becomes limiting. This would slow DNA replication overall, but it would not delay S-phase entry in lagging sister nuclei, as observed. Last, the localization of R to nuclei could be driven by active nuclear import rather than association with DNA. Although feasible, this mechanism would require nuclear cycle-dependent regulation of nucleocytoplasmic transport in addition to resource-DNA association; including this in a model appears unwarranted given the available data. Thus, plausible alternatives to the resource competition model are either ruled out by the experimental finding or appear less parsimonious.

Sequential DNA replication speeds up nuclear multiplication, but is maximal proliferation in RBCs in fact beneficial for *P. falciparum*? Assuming that faster nuclear multiplication leads to a higher merozoite number and/or shorter overall schizogony, it will increase the rate of proliferation of the parasite in the host’s blood. Overly fast proliferation of pathogens has been argued to imply rapid host mortality, which would reduce the chance for transmission and therefore pathogen fitness^56–58^. For malaria with an overall sub-1% mortality and a substantial fraction of mild or asymptomatic infections^59^, this so-called virulence-transmission trade-off does not appear dominant. Furthermore, at later stages of persistent disease, adaptive host immunity curtails RBC reinfection by merozoites. Therefore, maximizing the merozoite production rate becomes beneficial for continued transmission.

*P. falciparum* has evolved additional traits that strongly suggest a selective pressure for speedy schizogony. Because infected RBCs have altered mechanical properties, schizonts are at risk of being eliminated when passing with the bloodstream through the mechanical RBC quality control system in the spleen. To counter the risk, parasites induce a system of adhesive knobs on the RBC surface^60^ that increase the time between passages through the interendothelial slits in the spleen beyond the normal interval of around 5 h^61^. In combination with adhesion, rapid nuclear proliferation will then increase the chances of completing the proliferative cycle before being detected by the RBC quality control. Further speeding up proliferation, metabolic activity is increased by the production of parasite proteins that increase the permeability of RBCs to nutrients^62^.

In summary, *P. falciparum* appears to be under pressure for rapid proliferation in an environment that poorly supports parasite growth. Asynchronous nuclear cycles may be the evolutionary response to this challenge, by enabling economical use of the DNA replication machinery, with important benefits: First, by producing a minimal amount of DNA replication machinery, *P. falciparum* can divert the saved cellular supplies to other purposes. Second, *P. falciparum* can initiate nuclear multiplication when resource R is not yet abundant, which means that the cell cycle can proceed earlier. Both effects should reduce the total time to complete schizogony. To test for speed optimization, we compared simulations of model 3 using the best-fit, sequential-mode parameter set, to simulations with identical parameters except that *k*_*u*_, *k*_*b*_ were increased into the parallel-mode regime while fixing the dissociation constant *k*_*b*_/*k*_*u*_. We found that this single change caused nuclear multiplication to stay largely synchronous and to slow down by $34\min \simeq 7\%$ (Fig. 6g). This argues that resource usage in *P. falciparum* may in fact be optimized for maximally efficient proliferation within RBCs.

This work shows that competition for a limited resource is not necessarily an impairment that has to be overcome, but can be a mechanism to organize growth for maximum efficiency. This mechanism could also be relevant for other proliferating cells. While bacterial communities are well-known to secrete and share enzymes, it is unclear whether shared enzymes can intermittently localize to different bacterial cells. Multinucleated eukaryotic cells with nuclei in close proximity could share the DNA replication machinery between nuclei by diffusion, making them candidates for efficient resource competition. For instance, in spontaneously arising multinucleated HeLa cells, suboptimal levels of nutrients seemed to promote asynchrony^63^, potentially indicating competition among nuclei. The many nuclei in fungal hyphae are separated spatially by several *μ*m, which hinders the diffusive sharing of replication machinery. It is an interesting open question whether they could instead exploit periodic cytoplasmic flows for resource sharing, maximizing efficiency in a way similar to the mechanism described here. Interestingly, in *Aspergillus nidulans* hyphae, nuclear synchrony appearing under optimal growth conditions is lost depending on the sources of carbon and nitrogen in the culture medium^64^. In the early developing zebrafish embryo, cell cycles have been recently shown to slowly desynchronize dependent on the nucleocytoplasmic ratio set in the first division rounds^65^, which also suggests a limitation by a cytoplasmic resource. However, artificial syncytialization of the embryo couples nuclei more strongly, which has a synchronizing effect^65^. This indicates a global control of cycle timing similar to *Drosophila*, in contrast to the desynchronizing effect of resource competition we observe in *P. falciparum*.

While we have focused on asynchrony arising from competition for shared proteins involved in replication, S^*^-phases in *P. falciparum* also depend on other factors. For example, the kinase *Pf*CRK4 regulates a set of proteins involved in origin of replication firing^6,45^. In the context of our model, activity of *Pf*CRK4 may indicate or instruct S^*^-phase entry, while R controls processive replication (S-phase). In addition, how nuclear multiplication is embedded in the overarching rhythmicity of *Plasmodium* development inside an erythrocyte (a multiple of 24 h depending on the species^66^), remains elusive.

In conclusion, we have found that the observed asynchronous nuclear cycles during *P. falciparum* schizogony can be explained by competition for a shared replication resource, expanding the regulatory repertoire assigned to limiting pool mechanisms^38,39^. Competitive sharing allows *P. falciparum* to use the DNA replication machinery more efficiently, speeding proliferation, and increasing fitness in the hostile environment of the human host.

## Methods

### *P. falciparum* cell culture

The *P. falciparum* strain 3D7 is laboratory-adapted and is derived from the isolate NF54 by limiting dilution. NF54 originated from a case of airport malaria in the Netherlands^67^. *P. falciparum* 3D7 was cultivated in fresh O, Rh+ erythrocytes at 2−4% hematocrit in supplemented RPMI 1640 medium (with 0.2 mM hypoxanthine (CCPro), 25 mM HEPES, pH 7.4 (Merck), and 12.5 μg/ml gentamicin (Carl Roth), as well as 0.5% AlbuMAX II (Gibco)), at 37 ^∘^C in 90% relative humidity, 5% O_2_, and 3% CO_2_^68^. Routine synchronizations were performed by 5% sorbitol treatment as previously described^69^.

### *P. falciparum* cell lines

The PCNA1::GFP and NLS::mCherry expressing *P. falciparum* 3D7 line was previously generated^13^. To detect DNA replication events, PCNA1::GFP was expressed from the episomal plasmid pARL_PCNA1-GS-eGFP under the control of the *P. falciparum* CRT promoter and the *P. berghei* DHFR/TS terminator. *P. falciparum* PCNA1 is connected to eGFP via a 2 × GGGGS-linker. Additionally, this plasmid contains a WR99210 resistance cassette^70^. To visualize the nucleoplasm and detect nuclear division events, mCherry coupled to three nuclear localization sequences (NLS) was expressed under the control of the *P. falciparum* hsp86 promoter and the *P. berghei* DHFR/TS terminator. Furthermore, this plasmid contains a blasticidin (BSD) resistance cassette.

### Live-cell imaging

For live-cell imaging, we followed previously published protocols with minor modifications^13,71^. Sterile glass-bottom 8-well dishes (ibidi GmbH) were coated with 5 mg/ml Concanavalin A (Merck) and rinsed with PBS. To generate a monolayer of cells, approximately 500 μl of resuspended parasite culture was washed twice with supplemented RPMI without AlbuMAX II and left to settle on the dish for 10 min at 37 ^∘^C before unattached cells were washed off using supplemented RPMI without AlbuMAX II. Cells were left to recover at standard culturing conditions in supplemented RPMI with AlbuMAX II for at least 8 h before media was exchanged to the phenol red-free imaging medium (RPMI 1640 L-Glutamine, PAN-Biotech, 0.5% AlbuMAX II, 0.2 mM Hypoxanthine, 25 mM HEPES pH 7.3, 12.5 μg/ml gentamicin), which either had been equilibrated to incubator gas conditions for at least 6 h before sealing of the imaging dish or imaging was conducted in a gassed incubation chamber. Unless otherwise noted, long-term live-cell imaging was carried out on a PerkinElmer UltraVIEW VoX microscope equipped with Yokogawa CSU-X1 spinning disk head and Nikon TiE microscope body. An Apo TIRF 60×/1.49 N.A. oil immersion objective and Hamamatsu C9100-23B EM-CCD camera were used. Live-cell imaging was performed at 36.5 ^∘^C. Images were acquired at multiple positions using an automated stage and the Perfect Focus System (PFS) for focus stabilization with a time resolution of 5 min/stack. Multichannel images were acquired sequentially using solid-state lasers with excitation at 488 nm and 561 nm and matching emission filters in addition to differential interference contrast (DIC) images; 8 μm stacks were acquired with a *z*-spacing of 500 nm.

For an independent data set, we used point laser scanning confocal microscopy, performed on a Zeiss LSM900 microscope equipped with an Airyscan 2 detector using Plan-Apochromat 63×/1,4 oil-immersion objective. Live-cell imaging was performed at 37 ^∘^C. The images were acquired using the Airyscan detector in SR mode at multiple positions using an automated stage and the Definite Focus module for focus stabilization with a time resolution of 5 min/stack for up to 18 h. Multichannel images were acquired sequentially using 488 nm and 561 nm diode lasers for eGFP and mCherry imaging, respectively. Emission detection was configured using variable dichroic mirrors to be 490−570 for eGFP and 570−650 for mCherry detection. The Airyscan detector was used with the gain adjusted between 700 and 900 V, offset was not adjusted (0%). Brightfield images were obtained using a transmitted light PMT detector, with the gain adjusted between 300 and 500 V. The sampling was Nyquist-optimized in *xy* axis (approx. 50 nm), and 600 μm in *z* axis, bidirectionally with pixel dwell time around 0.7 μs. Subsequently, the ZEN Blue 3.1 software was used for 3D Airyscan processing with automatically determined default Airyscan Filtering (AF) strength.

### Analysis of live-cell imaging data

The initial processing and analysis of the imaging data were done with Fiji^72^. For further analysis, we used Microsoft Excel. Multiposition images were manually inspected for dead or abnormal parasites using the DIC or brightfield channel. Individual data were saved as either 200 × 200 or 300 × 300-pixel TIFF files containing original channel, time and *z*-slice information. The time-lapse images were stabilized in *xy* as previously described^13^. In brief, using the Register Virtual Stack Slices Plugin in Fiji, we registered for each parasite a time-lapsed reference *z*-slice of the brightfield channel via the translation mode (no deformation) at standard parameters. The transformation matrices were saved and applied to all other *z*-slices and channels using the Transform Virtual Stack Slices Plugin in Fiji. Alternatively, we used the Fiji “Correct 3D Drift” plug-in^73^, which corrects for movement in the *xy* plane within a specified maximum pixel shift, using a reference channel (here, brightfield).

The fluorescence intensity over time was previously published and re-analyzed as shown in Supplementary Fig. 4a, b^13^. In brief, to reduce phototoxicity, we selected cells for long-term imaging, which showed a substantial PCNA1::GFP fluorescent signal around the onset of the first S-Phase. For analysis of the total signal intensity over time, we selected cells where egress was clearly visible as a reference time point for alignment. During imaging, z-stacks of small regions of interest centered around the parasite were acquired. To quantify the total signal intensity of our reporter proteins within these parasites over time, we collapsed all z-slices of a stack for each time point via average intensity z-projection, measured the raw integrated density of these average intensity projections, and subtracted the background signal. Finally, we normalized the signal to the maximum signal per series to be able to compare parasites showing different protein levels and aligned the traces to the time point of egress.

We determined the dynamics of the nuclear cycle phases using our nuclear cycle sensor line and as previously described^13,45^. In brief, S-phase was defined as the time interval between the onset and the end of visible PCNA1::GFP accumulation in a given nucleus. The interval between the end of the visible PCNA1::GFP accumulation and the ensuing accumulation was defined as division (D-) phase.

To quantify the number of nuclei in a given *P. falciparum* schizont (Supplementary Fig. 2), we manually counted the number of mCherry-positive structures in each cell over time. Similarly, to determine the number of nuclei in S-phase at any given time point (Fig. 6f), we counted the number of mCherry-positive structures that also exhibited an accumulated PCNA1::GFP signal within the same cell. For image visualization and quantification of nuclei and nuclei in S-phase, we used the Imaris software (Oxford Instruments), versions 10.1 and 10.2.

### Uncoupled models 1 and 2

#### Independent nuclei

In model 1, each nuclear phase is an independent stochastic time span, and all nuclei are statistically identical. Model 1, therefore, is fully specified by the distributions *p*(*τ*_D_) and *p*(*τ*_S_) of D- and S-phase durations, respectively. We extracted these distributions from data in the 1- and 2-nuclei stages and used the empirical distributions to draw independent D- and S-phases. See also Supplementary Note 1.

#### Mother–daughter inheritance

Model 2 extends model 1 by including a statistical dependence among mother and daughter nuclei. Nuclear factors that influence the mother phase durations and, after inheritance, the daughter phases, generate mother-daughter and daughter–daughter (sister) correlations. The sister correlations can be further modified. First, factors that do not affect the mother phases, for instance because they are produced late in the nuclear cycle, can correlate the daughter phases. Second, D-phases implicitly contain a shared initial period from the end of the mother S-phase to nuclear division, which is expected to contribute positive correlations between sister D-phases. In technical terms, these effects generate partial correlations between sisters given the mothers.

All dependencies are captured by the joint distribution of the six mother and daughter D- and S-phases, which specifies model 2 as a bifurcating auto-regressive (BAR) process^29^ for the nuclear phase durations. To calibrate model 2, we first transform the experimental phase durations onto standard normal distributions. E.g., the normal variate *x*_*d*_ for the mother D-phase is obtained as ${x}_{d}=G({\tau }_{{{{\rm{D}}}}})={C}_{{{{\rm{gauss}}}}}^{-1}({C}_{{{{\rm{D}}}}}({\tau }_{{{{\rm{D}}}}}))$, where *C*_gauss_, *C*_D_ denote the normal and data-derived empirical cumulative distribution functions, respectively. The covariance matrix **Σ** between the six standardized phase durations is known as the Gaussian-rank correlation matrix of the measured phase durations. Model 2 was then calibrated using our experimental data from stages 1–4 to determine **Σ**; we included all statistically significant correlations and set insignificant ones to zero (Fig. 2c, d; Supplementary Fig. 1 and Supplementary Note 2 for details). Model 2 then generated nuclear lineage trees by first recursively sampling Gaussian lineage trees according to **Σ** and then back-transforming to experimental time via the inverse map *G*^−1^.

### Resource sharing model 3

We complete the qualitative description of model 3 given in the main text by stating and discussing the defining equations. Table 1 summarizes the symbols used. In *P. falciparum*, licensed origins of replication are abundant^5,74^, and the overall DNA replication rate in a nucleus is limited by the number of active replication forks (initiated at licensed and activated origins). In the model, we consider inactive/partial replication forks and active replication forks. For simplicity, we take the total number of replication forks *f*_*i*_ in any S^*^-phase nucleus to be identical and measure all molecule numbers relative to *f*_*i*_. Then in any S^*^-phase nucleus *i* we have $1\equiv {f}_{i}={f}_{i}^{{{{\rm{free}}}}}+c_i$, where ${f}_{i}^{{{{\rm{free}}}}}$ designates the amount of inactive forks F_*i*_ without loaded resource, and *c*_*i*_, the amount of active resource-fork complexes RF_*i*_, respectively. In D^*^-phase nuclei no active forks exist, and ${f}_{i}^{{{{\rm{free}}}}}=c_i=0$ (Fig. 3a, b). Summing over all nuclei, ${f}^{{{{\rm{free}}}}}\equiv {\sum }_{i}{f}_{i}^{{{{\rm{free}}}}}$ and *c* ≡ ∑_*i*_*c*_*i*_, respectively, we then find that ${f}^{{{{\rm{free}}}}}+{c}={n}_{{{{\rm{S}}^{*}}}}$, the number of nuclei in S^*^-phase at any given time.

**Table 1:** Overview of symbols used in model 3

Symbol	Definition	Unit
*f*_*i*_	total replication forks in nucleus *i*, ${const.}\equiv 1$	*f*_*i*_
${f}_{i}^{{{{\rm{free}}}}}$	inactive replication forks in nucleus *i*	*f*_*i*_
*c*_*i*_	activated replication forks in nucleus *i*	*f*_*i*_
$c_{i,\min}$	detection threshold for active replication forks	*f*_*i*_
${n}_{{{{\rm{S}}}}}^{*}$	number of S^*^-phase nuclei	1
*f*^free^	inactive replication forks in the cell	*f*_*i*_
*c*	activated replication forks in the cell	*f*_*i*_
*r*^free^	total free resource in the cell	*f*_*i*_
*r*	total resource in the cell	*f*_*i*_
*k*_*u*_	RF unbinding rate	${\langle {\tau }_{\min }\rangle }^{-1}$
*k*_*b*_	binding rate	${f}_{i}^{-1}{\langle {\tau }_{\min }\rangle }^{-1}$
*g*_*h*_	DNA content of one haploid genome, ${const.}\equiv 1$	*g*_*h*_
*g*_*i*_	DNA content in nucleus *i*	*g*_*h*_
*g*	DNA content in the cell	*g*_*h*_
${\dot{g}}_{i}$	DNA synthesis rate in nucleus *i*	${g}_{h}{\langle {\tau }_{\min }\rangle }^{-1}$
*ρ*	unlimited DNA synthesis rate in a nucleus	${g}_{h}{\langle {\tau }_{\min }\rangle }^{-1}$
*τ*	nuclear cycle duration	$\langle {\tau }_{\min }\rangle$
*τ*_*X*_	phase duration of phase *X*	$\langle {\tau }_{\min }\rangle$
$\langle {\tau }_{\min }\rangle$	mean unlimited cycle duration, ${const.}\equiv 1$	$\langle {\tau }_{\min }\rangle$
*ζ*	resource availability	1
*ζ*_*c*_	critical availability for unlimited growth	1
*η*	resource utilization	1
*λ*	steady growth rate	${\langle {\tau }_{\min }\rangle }^{-1}$
${\lambda }_{\max }$	unlimited steady growth rate	${\langle {\tau }_{\min }\rangle }^{-1}$

In a similar fashion, we also introduce the amounts of free resource *r*^free^ and total resource *r* ≡ *r*^free^ + *c*. In our units, *r* equals the maximum number of nuclei that could be fully engaged in active DNA replication with the available resources at any given time. When available forks are abundant, we expect *r* = *c* with no free resource remaining, *r*^free^ = 0.

With these definitions, mass-action kinetics for resource loading and unloading in nucleus *i* (Eq. (1)) read

7 $$\dot{c}_{i}={k}_{b}{f}_{i}^{{{{\rm{free}}}}}{r}^{{{{\rm{free}}}}}-{k}_{u}c_i={k}_{b}(1-c_i)(r-{\sum }_{j}c_j)-{k}_{u}c_i.$$

This is a system of coupled ordinary differential equations for the set of active forks {*c*_*i*_}, driven by the time-varying total resource *r*. The coupling arises via the shared pool *r*^free^ of free resources for which S^*^-phase nuclei compete (Fig. 3c). The lifetime of active forks is set by the inverse rate constant ${k}_{u}^{-1}$. The timescale for fork activation is set (in the resource-rich regime) by ${(r{k}_{b})}^{-1}$.

The DNA synthesis rate ${\dot{g}}_{i}$ in nucleus *i* is taken to be proportional to the number *c*_*i*_ of activated forks:

8 $${\dot{g}}_{i}=\rho {{c}}_{i}\,.$$

This makes the assumption that replication forks do not interact, e.g., by competing for nucleotides^75^. The DNA amount *g*_*i*_ increases from 1 to 2 haploid genomes during S^*^-phase. Because the time point of nuclear division is not resolved in the model, for simplicity, *g*_*i*_ is reset to 1 at the start of each daughter D^*^-phase. The total DNA amount *g* ≡ ∑_*i*_*g*_*i*_ increases continuously with time^6,45^. A fully activated nucleus (*c*_*i*_ = 1) is seen to replicate at a maximal rate *ρ*, so that 1/*ρ* can be recognized as the minimum possible S-phase duration in the model.

Finally, motivated by the shape of observed D-phase durations, D^*^-phases are modeled as Gamma-distributed, parameterized by the mean $\langle {\tau }_{{{{\rm{D}}^{*}}}}\rangle$ and standard deviation ${\sigma }_{{{{\rm{D}}^{*}}}}$. In resource-rich conditions, the cycle time is given by

9 $$\tau={\tau }_{{{{\rm{S}}^{*}}}}+{\tau }_{{{{\rm{D}}^{*}}}}\to {\tau }_{\min }=1/\rho+{\tau }_{{{{\rm{D}}^{*}}}},$$

and it follows shifted a Gamma distribution with mean $\langle {\tau }_{\min }\rangle=1/\rho+\langle {\tau }_{{{{\rm{D}}^{*}}}}\rangle$ and standard deviation ${\sigma }_{{{{\rm{D}}^{*}}}}$. We use the mean unlimited cycle duration $\langle {\tau }_{\min }\rangle$ as the base unit for measuring time. In resource-limited conditions, the average cycle time is increased by slowed DNA replication.

To model experimentally observed S- and D-phases, we assign a nucleus *i* to S-phase when activated forks exceed a detection threshold $c_{i,\min} \approx 15\%$. Nuclei not in S-phase are assigned to D-phase.

### Steady-growth rate and resource utilization

To determine the steady-growth values of *η* and *λ*, first consider resource-limited cells (i.e., $r < {n}_{{{{\rm{S}}^{*}}}}$). High RF binding affinity ensures that resource is fully bound to forks at all times, i.e., *η* = 1. Eq. (5) takes on the constant value *λ* = *ζρ*. Conversely, in non-resource limited cells where $r > {n}_{{{{\rm{S}}^{*}}}}$, resource fully saturates available forks. Thus, every S^*^-phase nucleus *i* has *c*_*i*_ = 1 and completes S^*^-phase after the minimal duration 1/*ρ*; the full nuclear cycle takes $\tau=1/\rho+{\tau }_{{{{\rm{D}}^{*}}}}$ (Eq. (9)). As a result, the population growth rate attains its maximal value ${\lambda }_{\max }$, given implicitly by the Malthusian relation (e.g., ref. ^46^, see also Supplementary Note 5)

10 $${e}^{-{\lambda }_{\max }/\rho }\int_{0}^{\infty }p({\tau }_{{{{\rm{D}}^{*}}}}){e}^{-{\lambda }_{\max }{\tau }_{{{{\rm{D}}^{*}}}}}\,d{\tau }_{{{{\rm{D}}^{*}}}}=\frac{1}{2},$$

from which we obtain $\eta={\lambda }_{\max }/(\rho \zeta )$ by Eq. (5).

Using the definitions Eqs. (3), (5) and the Malthusian relation, Eq. (10), we can summarize the steady-growth limit as

11 $$\lambda=\min (\zeta \rho,{\lambda }_{\max }),\quad \eta=\lambda /(\zeta \rho ),$$

with ${\lambda }_{\max }$ given by Eq. (10). In the limit of deterministic ${\tau }_{{{{\rm{D}}^{*}}}}$, we can solve Eq. (10) explicitly to obtain

12 $$(\lambda,\eta )\to \left\{\begin{array}{ll}\left(\zeta \rho,1\right)&\zeta < {\zeta }_{c},\\ \left({\zeta }_{c}\rho,{\zeta }_{c}/ \zeta\right)&\zeta \ge {\zeta }_{c}\end{array}\right.,{{{\rm{where}}}}\,{\zeta }_{c}=\frac{\log 2}{1+{\tau }_{{{{\rm{D}}^{*}}}}\rho }.$$

We remark that Eq. (10) is invariant under shifts of $p({\tau }_{{{{\rm{D}}^{*}}}})$ and 1/*ρ* by equal and opposite amounts, which do not change the cycle time distribution. This explains the observation that in resource-saturated cells, the growth rate attains ${\lambda }_{\max }$ independent of S^*^-phase fraction.

### Extension of model 3 to match experimental data

To match experimental data from time-lapse microscopy, we modified model 3 to represent the dynamics of *P. falciparum* nuclear multiplication more realistically.

#### Resource abundance time course

We modeled the time course of replication resource R available in a *P. falciparum* cell on measured temporal profiles of total PCNA1::GFP and total NLS::mCherry fluorescence intensity^13^. We preprocessed the data through the following steps. First, we excluded non-responding cells, i.e., cells with almost no growth, indicated by a total fold-change <1.25. Second, for each of the reporters, the raw data were aligned at the moment of egress. We set the egress time to $t=854\,\min$, corresponding to the experimentally observed average interval from the onset of the first S-phase to egress ^13^. Third, for each of the reporters, the raw data was normalized such that the 15th percentile of intensities after egress was mapped to 0 and the 85th percentile of maximum intensities was mapped to 1. In instances where the drop after egress was not recorded, we mapped the 15th percentile of intensities before egress to 0. A sigmoidal curve was least-square fitted according to (Supplementary Fig. 4a, b)

13 $$I(t)=[1+\exp (-k(t-{t}_{1/2}))]^{-1},$$

resulting in ${t}_{1/2}^{{{{\rm{PCNA1}}}}}=24\pm 6\,\min,{k}^{{{{\rm{PCNA1}}}}}=0.005\pm 0.0002\,{\min }^{-1}$, and ${t}_{1/2}^{{{{\rm{NLS}}}}}=180\pm 2\,\min$, ${k}^{{{{\rm{NLS}}}}}=0.01\pm 0.0002\,{\min }^{-1}$, respectively. The final *r*(*t*) = *ξ* *I*(*t*) used the average of the two best-fit parameter sets (${t}_{1/2}=102\,\min$ and $k=0.0078\,{\min }^{-1}$) for the two available reporter proteins, retaining one adjustable parameter for the final saturating abundance *ξ* = *r*(*∞*). The final abundance *ξ* was sampled per parasite from a uniform distribution with adjustable range (*ξ*_low_, *ξ*_high_). In effect, *r*(*t*) grows initially but saturates approximately halfway through the schizont stage, after  ≈ 3 rounds of multiplication (Supplementary Fig. 4c).

#### Termination of nuclear multiplication

To simulate full *P. falciparum* nuclear multiplication within the resource-sharing model 3, we need to specify when multiplication is stopped. Our previous result indicated that schizogony is likely terminated when a predetermined number of nuclei is reached, not after a predetermined time^13,52^. Thus, the extended model 3 prohibits further S-phase entries when a predetermined fixed number *n*_final_ of nuclei have commenced S-phases.

### Approximate Bayesian Computation (ABC)

To estimate model 3 parameters, we used ABC based on the first Wasserstein distance metric between the predicted and observed standardized three-dimensional joint distributions of sister S-phases, delays and D-phases. We employed an ABC Sequential Monte Carlo protocol using *N* = 10,000 parallel particles as implemented in Python package pyABC. Simulations were run until the acceptance rate dropped below 1% (Supplementary Fig. 5). The resulting MAP parameter values and 95% credible intervals are given in Table 2.

**Table 2:** Overview of MAP parameter values and 95% credible intervals for model 3

Parameter	MAP value	Credible interval [95%]	Unit
*k*_*b*_	20	[10, 78]	${\langle {\tau }_{\min }\rangle }^{-1}$
*k*_*u*_	0.004	[0.0001, 0.5]	${\langle {\tau }_{\min }\rangle }^{-1}$
*ρ*	4.8	[3.5, 5.6]	${g}_{h}{\langle {\tau }_{\min }\rangle }^{-1}$
$\langle {\tau }_{\min }\rangle$	88	[80, 103]	$\min$
${\sigma }_{{{{\rm{D}}^{*}}}}$	0.09	[0.05, 0.15]	$\langle {\tau }_{\min }\rangle$
*ξ*_low_	0.8	[0.6, 1.2]	*f*_*i*_
*ξ*_high_	1.5	[1.1, 2.3]	*f*_*i*_

### Reporting summary

Further information on research design is available in the Nature Portfolio Reporting Summary linked to this article.

## Supplementary information

Supplementary Information

Reporting summary

Transparent Peer Review file

## Data Availability

The live-cell tracking datasets generated during and analyzed in the current study are available under https://github.com/hoefer-lab/seq-resource-sharing-code.git^76^. All raw imaging data generated during and analyzed in the current study are available from the corresponding authors upon request.

## Code availability

The computer code used to analyze data and for model simulations is available under https://github.com/hoefer-lab/seq-resource-sharing-code.git^76^.
