# Supplementary material for: Competitive resource allocation drives asynchronous and rapid nuclear multiplication in the malaria parasite
Source: Nat Commun. 2026 Jul 27;17:7413. doi: 10.1038/s41467-026-75378-x (PMC13408688; doi:10.1038/s41467-026-75378-x)
Supplement: Supplementary file 1 — Supplementary Information [file 41467_2026_75378_MOESM1_ESM.pdf]

Supplementary Information

Competitive resource allocation drives asynchronous and rapid nuclear multiplication in the malaria parasite

Mathematical details of the nuclear multiplication models

Patrick Binder,[1,2] Aistė Kudulytė,[3] Severina Klaus,[3,4] Thomas Höfer,[1] Ulrich S. Schwarz,[2] Markus Ganter,[3,*] and Nils B. Becker[1,†]

[1] *Theoretical Systems Biology, German Cancer Research Center (DKFZ), Heidelberg, Germany*

[2] *Institute for Theoretical Physics and BioQuant, Heidelberg University, Heidelberg, Germany*

[3] *Center for Infectious Diseases - Parasitology, Medical Faculty, Heidelberg University, Heidelberg, Germany*

[4] *present address: Center for Infectious Diseases - Virology, Medical Faculty, Heidelberg University, Heidelberg, Germany*

(Dated: June 19, 2026)

Supplementary Note 1. CONSTRUCTION AND CALIBRATION OF MODEL 1: INDEPENDENT-PHASES MODEL

Model 1 is a minimal branching process for nuclear phases. Nuclei have two phases: S-phase, during which active DNA replication happens, and D-phase which extends from the end of S-phase in the mother nucleus to the start of the next S-phase in either daughter nucleus. The S-phases are detected experimentally by PCNA1::GFP localization, and we collect their empirical distribution $p(\tau_S)$. From the intervening times between S-phases we collect the empirical distribution for D-phase durations $p(\tau_D)$. Then for each nucleus, sample durations for both phases are drawn from the two distributions, and a lineage is simulated by adding successive daughters. In practice, lineages were only simulated to the 2-nuclei stage as that was sufficient to analyze the generated sister correlations.

Supplementary Note 2. CONSTRUCTION AND CALIBRATION OF MODEL 2: BIFURCATING AUTOREGRESSIVE MODEL

In order to test if the correlation patterns in *P. falciparum* nuclear lineage trees can be explained by simple local inheritance, we consider model 2. Model 2 is constructed as a bifurcating autoregressive process (BAR) that incorporates the local correlations between the D- and S-phases existing between mother and both daughter nuclei.

In a first step, we consider a BAR process for a state vector $\mathbf{x}$ of standardized cycle phase durations of the mother and both daughters, which obey Gaussian statistics, as follows. Given the correlations between mother and daughters, we can specify a joint multivariate Gaussian distribution with mean vector $\boldsymbol{\mu}$ and

* ganter@uni-heidelberg.de
† nils.becker@dkfz.de

covariance matrix $\boldsymbol{\Sigma}$,

$$p(\mathbf{x}) = \frac{1}{\sqrt{\det(2\pi\boldsymbol{\Sigma})}} \exp\left[-\frac{1}{2}(\mathbf{x}-\boldsymbol{\mu})^\intercal \boldsymbol{\Sigma}^{-1}(\mathbf{x}-\boldsymbol{\mu})\right]. \tag{1}$$

Simulation of the branching process forward in time then involves drawing the daughter D- and S-phases conditioned on the given mother D- and S-phases, in agreement with Eq. 1. To obtain the required conditional distribution, we partition the multivariate $\mathbf{x}$, its mean and covariance as

$$\mathbf{x} = \begin{bmatrix} \mathbf{x}_m \\ \mathbf{x}_d \end{bmatrix}, \quad \boldsymbol{\mu} = \begin{bmatrix} \boldsymbol{\mu}_m \\ \boldsymbol{\mu}_d \end{bmatrix} \text{ and } \quad \boldsymbol{\Sigma} = \begin{bmatrix} \boldsymbol{\Sigma}_{mm} & \boldsymbol{\Sigma}_{md} \\ \boldsymbol{\Sigma}_{dm} & \boldsymbol{\Sigma}_{dd} \end{bmatrix}, \tag{2}$$

where subscripts $\cdot_m$ and $\cdot_d$ correspond to the phases of the mother and the phases of both daughters, respectively. It's well-known that the conditional distribution of $\mathbf{x}_d$ given $\mathbf{x}_m = \overline{\mathbf{x}}_m$ is also Gaussian, with adjusted mean $\overline{\boldsymbol{\mu}}_d$ and adjusted covariance matrix $\overline{\boldsymbol{\Sigma}}_{dd}$, given by

$$\overline{\boldsymbol{\mu}}_d = \boldsymbol{\mu}_d + \boldsymbol{\Sigma}_{dm}\boldsymbol{\Sigma}_{mm}^{-1}(\overline{\mathbf{x}}_m - \boldsymbol{\mu}_m), \tag{3a}$$

$$\overline{\boldsymbol{\Sigma}}_{dd} = \boldsymbol{\Sigma}_{dd} - \boldsymbol{\Sigma}_{dm}\boldsymbol{\Sigma}_{mm}^{-1}\boldsymbol{\Sigma}_{md}. \tag{3b}$$

To forward simulate a standardized Gaussian version of a nuclear population, we begin from the founder nucleus' S-phase, as the preceding initial D-phase is not available from experiment. Thus, for the initial generation only, $\mathbf{x}_m$ is a one-dimensional standard Gaussian variable, while in subsequent generations, $\mathbf{x}_m$ is two-dimensional (covering both D- and S-phases). $\mathbf{x}_d$ is a four-dimensional standard Gaussian variable for the daughters' D- and S-phases.

In a second step we map this Gaussian bifurcating process onto the correct empiri-

cal phase distributions. Specifically, the empirical mother-phases, denoted by $\boldsymbol{\tau}_m$, are transformed into standard Gaussian variables $\mathbf{x}_m$ using the nonlinear mapping

$$\mathbf{x}_m = G(\boldsymbol{\tau}_m) = C_{\mathrm{gauss}}^{-1}\big(C_{\mathrm{ex}}(\boldsymbol{\tau}_m)\big) \, , \tag{4}$$

where C_{gauss} denotes the cumulative distribution function (CDF) of the standard Gaussian distribution and C_{ex} is the CDF corresponding to the empirical data. Conversely, simulation data matching the empirical phase distributions can be generated from Gaussian samples by the inverse mapping

$$\boldsymbol{\tau}_m = G^{-1}(\mathbf{x}_m). \tag{5}$$

The daughter phases are transformed in the same way.

Thus, to simulate nuclear populations in model 2, we first sample a standard Gaussian for the initial S-phase, then successively apply Eq. 3 to produce a Gaussian correlated tree, and finally transform onto the correct marginal distributions using Eq. 5.

The remaining task is to parametrize model 2, by specifying the empirical distributions C_{ex} and correlation matrix $\boldsymbol{\Sigma}$. We first study the empirical phase distributions assessing whether empirical distributions from related cells can be pooled to construct more robust cumulative distribution functions C_{ex} for the S- and D-phases.

As reported in [1], the very first S-phase is significantly prolonged compared to later S-phases. To account for this difference, model 2 uses this specific prolonged empirical distribution for the initial S-phase.

We next examine whether S-phases at the 2- and 4-nuclei stages follow a common distribution. Using the Kolmogorov-Smirnov (KS) test to compare each individual

phase distribution to the S-phase durations pooled across the two stages confirms that S-phases can be treated as independent samples from a common distribution (Supplementary Fig. 1a). Furthermore, later stages do not significantly deviate, given the scarce available data. Consequently, we model all S-phases starting from the 2-nuclei stage using the pooled distribution.

By convention, D-phases at the 2-nuclei stage are ordered such that the first sister nucleus always has a shorter D-phase than the second, i.e. $\tau_{\mathrm{D}_1} \leq \tau_{\mathrm{D}_2}$. Indeed, D_1-phases follow a distribution shifted to shorter times compared to D_2. This effect may either simply be an artifact of our ordering convention, or there may exist an intrinsic asymmetry in the nuclear division process that by itself already generates a faster and a slower D-phase. To scrutinize this possibility, we constructed a symmetrized distribution by drawing 50 000 independent pairs of D-phases from the pooled empirical D-phase distribution, assigning the faster value to D_1 and the slower value to D_2. Using the two-sample KS test, we find that the so-constructed D_1 and D_2-phase distributions are statistically indistinguishable from the corresponding empirical distributions (Supplementary Fig. 1a). We conclude that the data do not support asymmetric division, and model the 2-nuclei stage D-phases as symmetric and independent, using the pooled empirical distribution.

A similar analysis for the 4-nuclei stage revealed that the D-phases of all four sister and cousin nuclei can also be described as independent samples from a common distribution (Supplementary Fig. 1a). Therefore, we model the D-phases at the 4-nuclei stage using the pooled distribution of the empirical data from D_{11}, D_{12}, D_{21}, and D_{22}. Because no later D-phases were observed, we continue to use this pooled distribution to model all subsequent D-phases.

The final parametrized model 2 then comprises four empirical phase distributions in total. One S-phase and one D-phase distribution describe the initial phases, while two more distributions, pooled from later data, describe all subsequent S-and

D-phases, respectively (Supplementary Fig. 1b). To avoid introducing a censoring bias in pooling, we include a nuclear phase only if the corresponding phase was also observed in the sister nucleus.

We next describe our procedure for extracting correlations from data to obtain $\mathbf{\Sigma}$. First, we transform the empirical data into standard Gaussian variables using Eq. 4. The Gaussian rank correlations of the data then by definition equal the moment correlation coefficients between these transformed variables. Because the imposed ordering of sister D-phases ($\tau_{\mathrm{D}_1} \leq \tau_{\mathrm{D}_2}$) would introduce a bias towards positive correlation, and in accordance with symmetric divisions, we computed the sister Gaussian rank correlation of the D-phases with shuffled pairs. Based on the estimated Gaussian rank correlations, we then constructed a parsimonious covariance matrix that retains the significant mother-daughter and sister correlations (two-sided p-value) but sets all non-significant correlations to zero. Bootstrapping confirmed that the common covariance Gaussian rank correlation matrix $\mathbf{\Sigma}$ is sufficient to capture the significant correlations across the different nuclear stages (Supplementary Fig. 1c). Explicitly, denoting $\boldsymbol{\tau} = \left[\tau_{\mathrm{D}}\ \tau_{\mathrm{S}}\ \tau_{\mathrm{D}_a}\ \tau_{\mathrm{D}_b}\ \tau_{\mathrm{S}_a}\ \tau_{\mathrm{S}_b}\right]^{\mathsf{T}}$ ($\cdot_a$ and $\cdot_b$ denote the two daughters), we obtain the Gaussian rank correlation matrix

$$\mathbf{\Sigma} = \left\langle g(\boldsymbol{\tau}) g(\boldsymbol{\tau})^{\mathsf{T}} \right\rangle = \left(\begin{array}{cc|cccc} 1 & 0 & 0 & 0 & 0 & 0 \\ 0 & 1 & 0 & 0 & 0.38 & 0.38 \\ \hline 0 & 0 & 1 & 0.45 & 0 & 0 \\ 0 & 0 & 0.45 & 1 & 0 & 0 \\ 0 & 0.38 & 0 & 0 & 1 & 0.79 \\ 0 & 0.38 & 0 & 0 & 0.79 & 1 \end{array}\right). \tag{6}$$

This matrix was used directly for multivariate Gaussian sampling according to Eqs. 3.

Supplementary Note 3. SEQUESTRATION OF RESOURCE

Sum Eq. 7 in the main text over i to obtain at saturation

$$0 \simeq \dot{c} = k_b n_{\mathrm{S}^*} r^{\mathrm{free}} - (k_u + k_b r^{\mathrm{free}})(r - r^{\mathrm{free}}), \tag{7}$$

which yields a free fraction $r^{\mathrm{free}}/r = \frac{1}{n_{\mathrm{S}^*} - r}\frac{k_u}{k_b} + \mathcal{O}(\frac{k_u}{k_b})^2$. At high affinity, this is $\ll 1$, as claimed.

Supplementary Note 4. CRITICAL AVAILABILITY

Because nuclei carry at least one genome, assuming $\zeta \geq 1$ in Eq. 2 in the main text implies that $r \geq n \geq n_{\mathrm{S}^*}$, which allows full rate of replication in all nuclei. This bound is not sharp: Most of the time, some fraction of nuclei is in D*-phase and does not compete for resource, so that a critical resource availability < 1 suffices for full speed replication. The critical value will decrease with increasing D*-to-S*-phase ratio.

Supplementary Note 5. POPULATION GROWTH RATE

To derive the population growth rate, consider a distribution of interdivision times τ with division time density $p(\tau)$ and survival function $S(a) = \mathbb{P}[\tau > a]$ valid for any newborn individual. After steady growth has been achieved, the average population $n(t)$ grows exponentially at (unknown) rate λ. The average total rate of divisions at time t satisfies

$$\lambda n(t) dt = n(t) \int_0^t \mathbb{P}[\text{age } a \mid \text{alive at } t]\mathbb{P}[\text{division in } (t, t+dt) \mid \text{age } a \text{ at } t] da. \tag{8}$$

The division propensity conditioned on survival to age a is given by

$$\mathbb{P}[\text{ division in } (t, t+dt) \mid \text{age } a \text{ at } t]/dt = -\dot{S}(a)/S(a). \tag{9}$$

The stationary age distribution can be obtained using Bayes' rule, by considering a base ensemble of all individuals born between 0 and t:

$$\begin{aligned}
&\mathbb{P}[\text{ age } a \mid \text{alive at } t]da = \\
&= \mathbb{P}[\text{ born in } (t-a-da, t-a) \mid \text{alive at } t] \\
&= \frac{\mathbb{P}[\text{ alive at } t \mid \text{born in } (t-a-da, t-a)]\mathbb{P}[\text{ born in } (t-a-da, t-a)]}{\mathbb{P}[\text{ alive at } t]} \\
&= \frac{S(a)\, e^{\lambda(t-a)}da/\int_0^t e^{\lambda(t-a')}da'}{\int_0^t S(a)\, e^{\lambda(t-a)}/\int_0^t e^{\lambda(t-a')}da'da} \\
&= \frac{S(a)e^{-\lambda a}da}{\int_0^t S(a')e^{-\lambda a'}da'}.
\end{aligned} \tag{10}$$

Here on the fourth line, we used the fact that births occur at a rate proportional to the exponentially growing population in the steady growth regime. Combining, Eq. 8 becomes

$$\begin{aligned}
\lambda &= \frac{-\int_0^t \dot{S}(a)e^{-\lambda a}da}{\int_0^t S(a')e^{-\lambda a'}da'} \\
&= \frac{-\lambda\int_0^t S(a)e^{-\lambda a}da - [S(a)e^{-\lambda a}]_0^t}{\int_0^t S(a')e^{-\lambda a'}da'} \\
&\to -\lambda + \frac{1}{\int_0^\infty S(a')e^{-\lambda a'}da'},
\end{aligned} \tag{11}$$

where we have integrated by parts and taken the limit $t\lambda \gg 1$. Solving Eq. 11 for the term appearing in the denominator yields $1/(2\lambda)$ for the denominator. Then

using $p(\tau) = -\dot{S}(\tau)$, we obtain

$$\int_0^\infty p(\tau) e^{-\lambda\tau} d\tau = \frac{\lambda}{2\lambda} = \frac{1}{2}. \tag{12}$$

Substituting $p(\tau) = p(\tau_{\mathrm{D}^*})$ where $\tau = \tau_{\mathrm{S}^*} + \tau_{\mathrm{D}^*}$ for the minimal $\tau_{\mathrm{S}^*} = 1/\rho$, this is seen to imply Eq. 10 in the main text.

Supplementary Note 6. SIMULATION DETAILS FOR MODEL 3

To characterize the asymptotic growth regime of model 3, we simulated nuclear multiplication until the lineage tree contained $2^{15} - 1$ nuclei entering S*-phase, corresponding approximately to 2^{14} terminal nuclei. This simulation depth proved sufficient to reach and maintain the steady-growth regime, as evident from Fig. 4. To extract the steady growth rate λ, we determined the steady-state nuclear cycle duration τ_∞ as the time required for a nucleus to complete a full cycle (D*-phase followed by S*-phase) under steady-state conditions. (In the limit of deterministic τ_{D^*} and $1/\rho + \tau_{\mathrm{D}^*} = \tau_{\min} = 1$, the expression for τ_∞ reads $\tau_\infty = \min\big(\log 2/(\zeta\rho), 1\big)$.) We then fitted an exponential function to the total DNA content g over approximately the final three cycles of the simulated data.

Similarly, the steady resource utilization η was obtained as the average of c/r across the final three cycles of simulations. The growth advantage $g_{\mathrm{seq}}/g_{\mathrm{par}}$ was read off at the end of the simulation, where it had converged sufficiently.

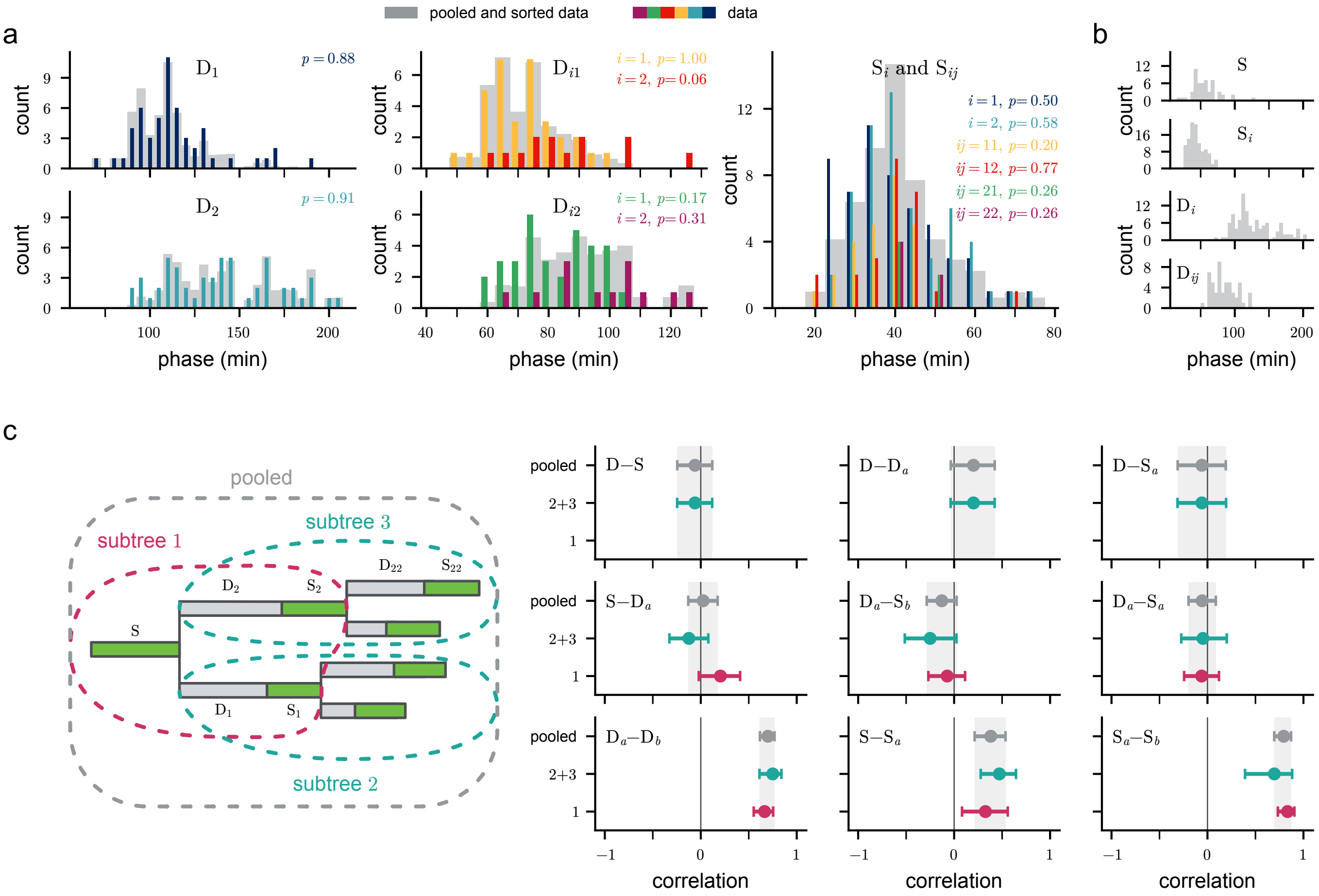

Supplementary Fig. 1. Overview of model 2 construction from empirical phase distributions and correlations. (a) Distributions of D-phase durations (D_i, D_{ij}) and S-phase durations (S_i, S_{ij}) at the 2- and 4-nuclei stages, demonstrating that each phase type can be described by a single pooled distribution. Two-sided p-values: two-sample Kolmogorov-Smirnov tests. (b) Pooled empirical distributions for S- and D-phases, which are used as input for model 2. (c) Left: Schematic of analyzed subtrees. Right: Gaussian rank correlations (mother-daughter and sister correlations) for all three subtrees, showing that a common correlation matrix accurately describes nuclear lineages.

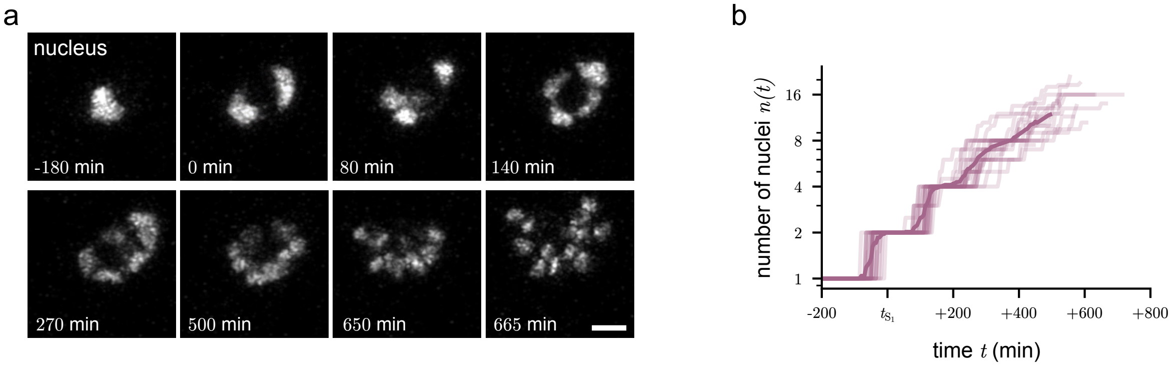

Supplementary Fig. 2. *P. falciparum* nuclear multiplication dynamics. (a) Time-lapse microscopy of the reporter parasite expressing 3xNLS::mCherry using a Zeiss LSM900 equipped with an Airyscan 2 detector; shown are maximum intensity projections of the mCherry channel; scale bar, 2 µm. (b) Number of nuclei in a *P. falciparum* schizont over time. Initial nuclear multiplication of *P. falciparum* is close to exponential. Traces from $N = 24$ cells were aligned at the start of S_1: Light: single trace. Dark: mean. Counts are reliable up to $t \approx 400$ min and approximate afterwards. 4 traces where tracked until egress, the remaining are truncated.

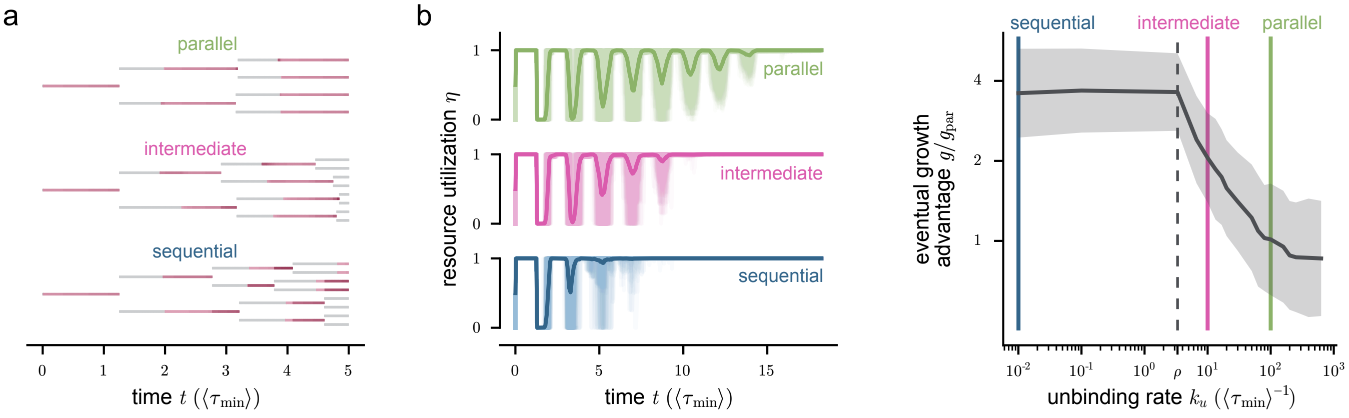

Supplementary Fig. 3. Growth advantage across replication modes, from sequential to parallel. (a) Example lineage trees for parallel, intermediate, and sequential replication modes. (b) Time course of resource utilization η for each mode. Lines, shading: simulation mean and single realizations, respectively. (c) Maximal eventual growth advantage g/g_{par}, occurring at $\zeta = \log(2)/\rho$ (cf. Fig. 5c), as a function of the unbinding rate k_u. k_u interpolates from sequential (low k_u, blue) over intermediate (magenta) to parallel (high k_u, green) replication regimes. Line, shading: simulation mean and standard error, respectively. Dashed line: unlimited DNA replication rate. Parameters as in Fig. 4; $\rho = 3.33$.

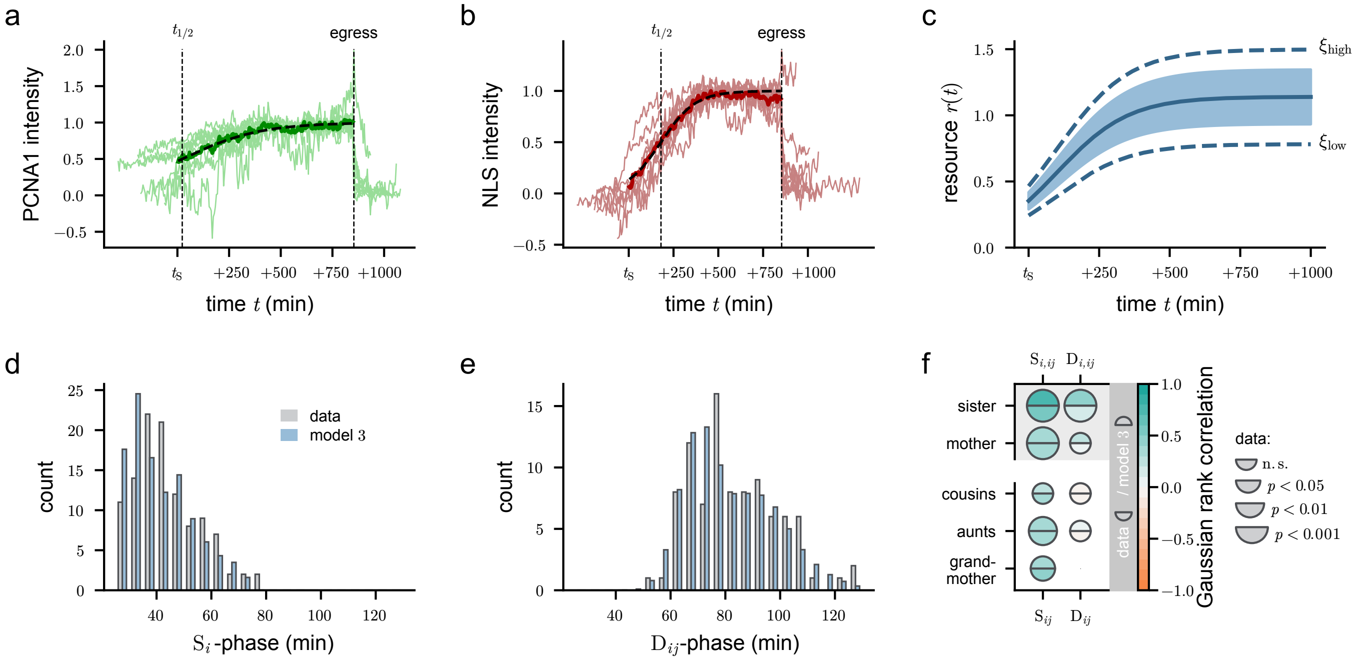

Supplementary Fig. 4. Overview of resource dynamics used and nuclear cycle dynamics produced in model 3. (a) Rescaled total PCNA1::GFP-fluorescence intensity ($N = 9$, see [1] Fig. S4C). (b) Rescaled total 3xNLS::mCherry-fluorescence intensity ($N = 11$, see [1] Fig. S2C). In ab, traces are aligned to egress and rescaled such that the intensity after egress maps to 0 and the maximum intensity maps to 1. For this rescaling, a moving window median was used. Light, dark, dashed lines: single traces, mean and least square fit, respectively. (c) Parametrized resource time course in model 3, resulting as an average over the fits in a and b. Solid Line, shading and dashed lines: mean, standard error and possible range of $r(t)$ realizations due to variability in ξ, respectively. (d) Distribution of S_i-phase durations: data vs. model 3. (d) Distribution of D_{ij}-phase durations: data vs. model 3. (f) Correlation structure. Bottom-half disks: data (with p-value). Top-half disks: model 3. Parameters as in Fig. 6.

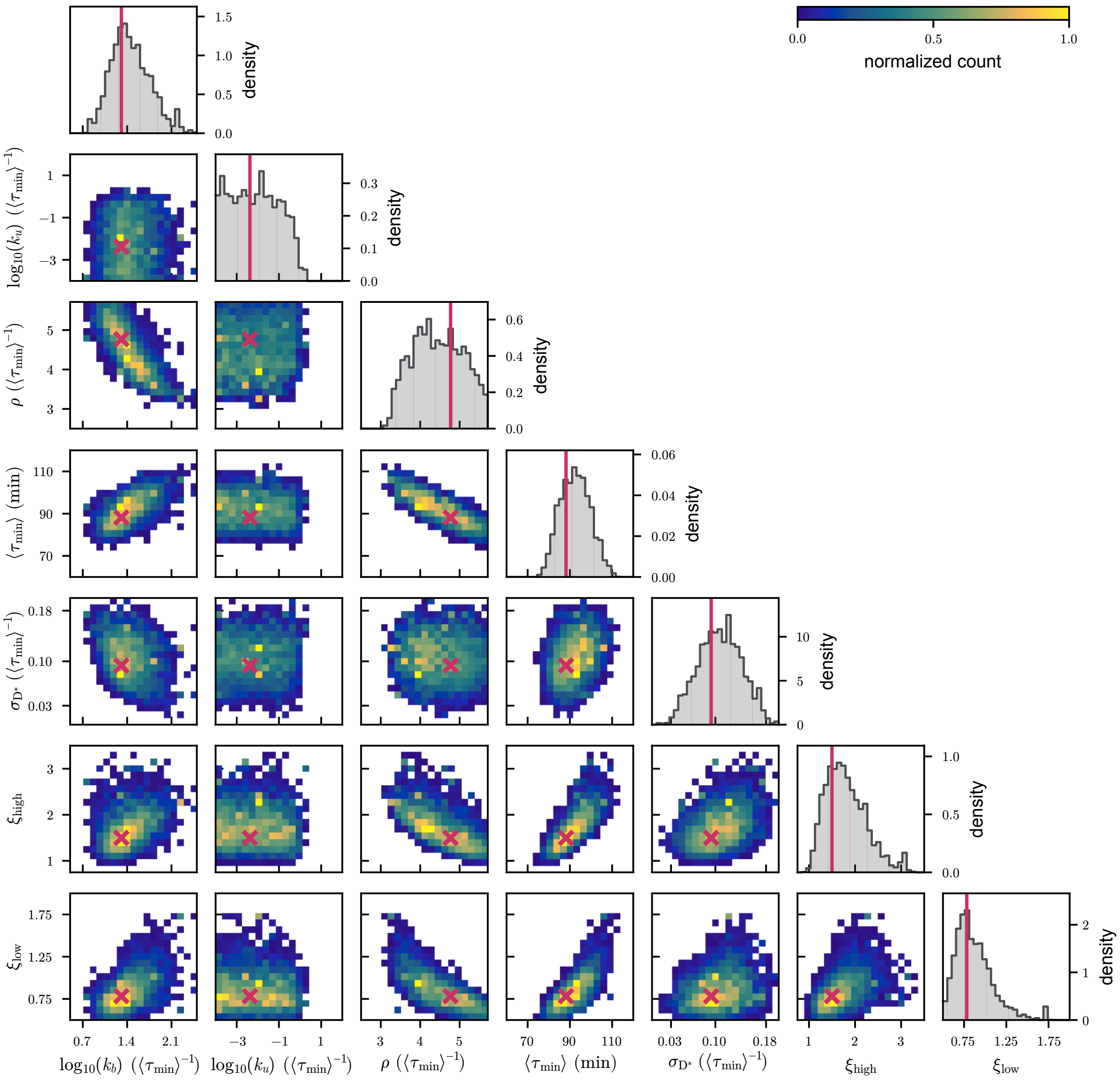

Supplementary Fig. 5. ABC parameter inference of model 3. (Sub)diagonal panels in the corner plot show the (2D)1D marginal posterior distributions. All parameters are unimodal, except for k_u, with an upper bound indicating the sequential replication mode. Red vertical lines or red crosses indicate the maximum a posteriori (MAP) estimate, obtained by maximizing a Gaussian kernel density estimate on the full-dimensional posterior distribution. All parameters were assigned uniform priors corresponding to the shown parameter range.

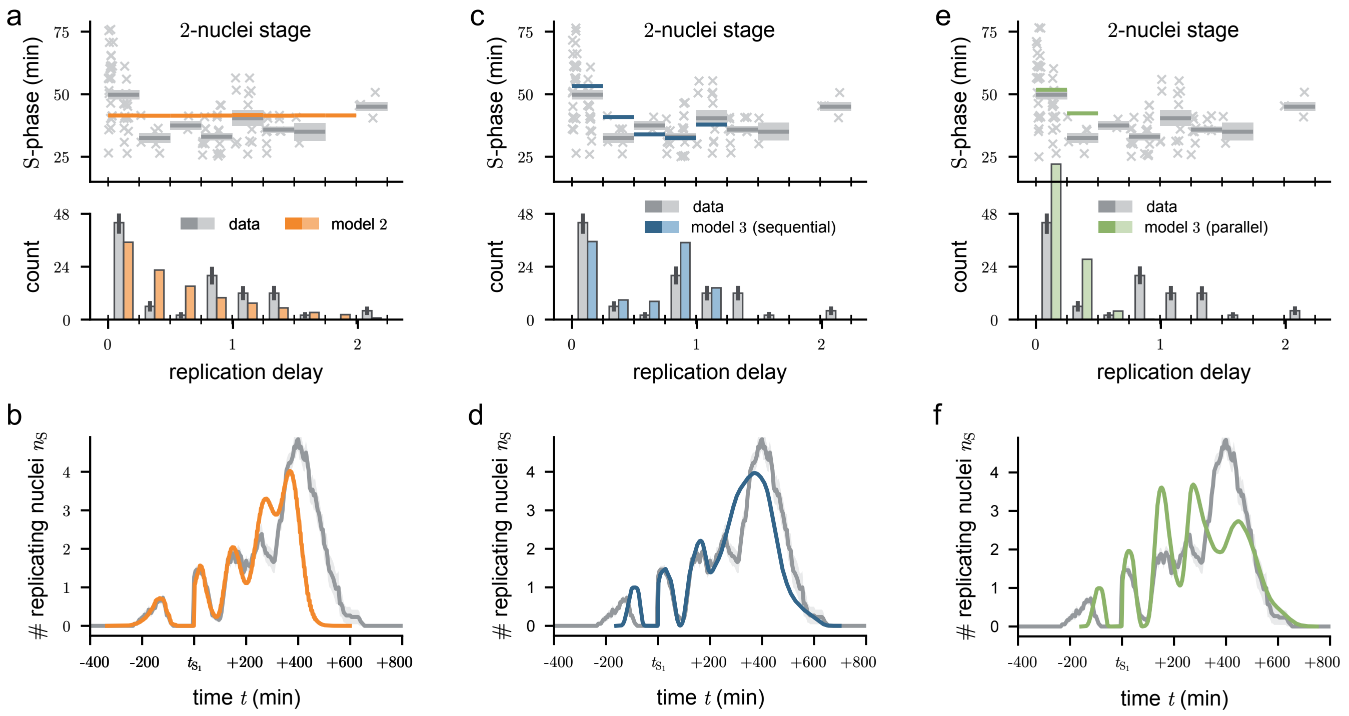

Supplementary Fig. 6. Comparison of model 2 (inheritance-based, uncoupled nuclei) and model 3 (resource-sharing, coupled nuclei) in reproducing key features of nuclear multiplication dynamics. (ace) Duration of S-phase versus replication delay (cf. Fig. 2f). (bdf): Temporal profiles of the number of replicating nuclei (cf. Fig. 6f). Gray: experimental data. In ab, orange: model 2. In cd, blue: best-fit model 3 in sequential mode. In ef, green: model 3 in parallel mode. This comparison highlights that only model 3 with sequential replication can reproduce both the observed asynchrony and the correlation between replication timing and duration.

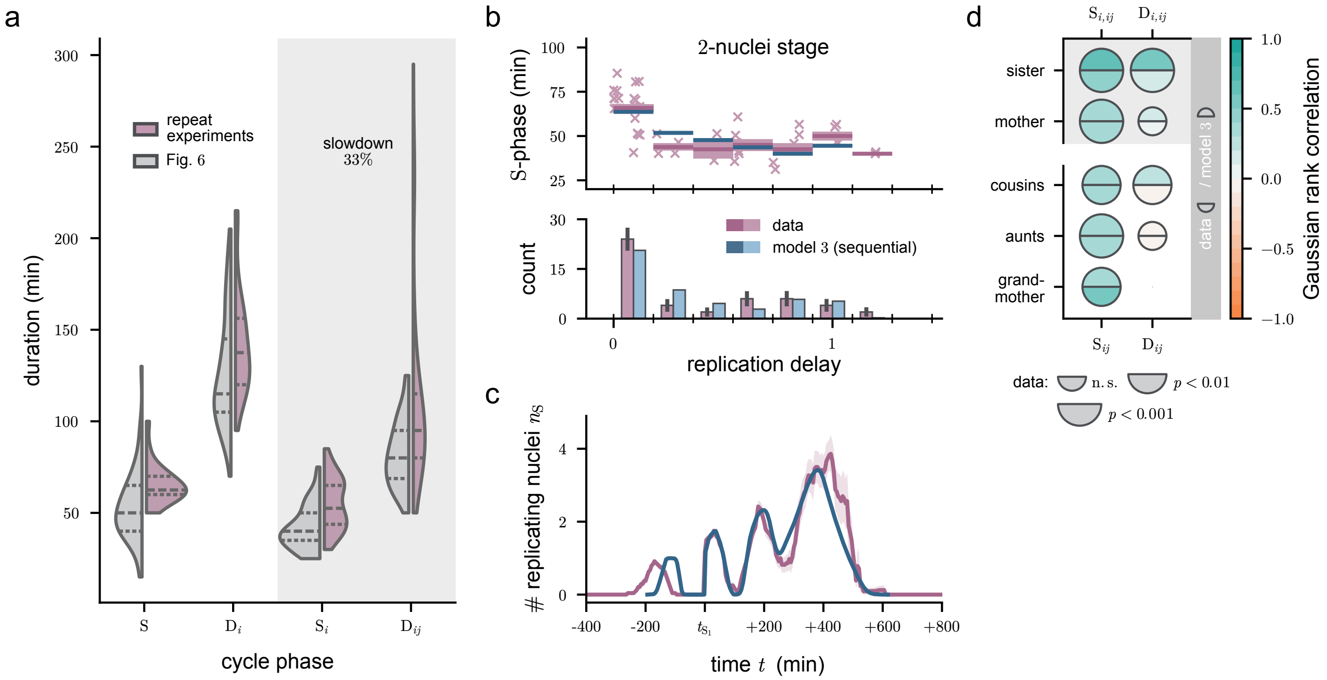

Supplementary Fig. 7. Model 3 captures the nuclear multiplication dynamics of *P. falciparum* schizonts observed in the repeat experiment on the Zeiss Airyscan microscope. (a) The initial two S- and D-phase durations are overall slower in the repeat experiment. Gray: data from main text; red: repeat experiment. As the two initial phases S, D_i are systematically longer in all conditions [1], we use S_i and D_{ij} (highlighted in light gray) to estimate the overall slowdown for the remainder of nuclear multiplication. (b) S-phase duration vs. delay. The sequential-mode model 3 with slowed overall cycle timing $\langle\tau_{\min}\rangle$ (blue) captures prolonged and enriched simultaneous S-phases as well as depleted intermediate-delay S-phases (cf. Fig. 6a). Repeat experiment (red): $N = 48$. (c) The model also successfully reproduces the observed synchronous temporal profile of replicating nuclei. (d) Gaussian rank correlation structure as in Fig. 2d. In a-d, 10^5 simulation realizations were run, each stopping at 16 nuclei. In b-d, $\langle\tau_{\min}\rangle = 111$ min; All other parameters as in Fig. 6. In c, traces from $N = 24$ cells were aligned at the start of S_1, and averaged. 5 traces were tracked until egress; the remaining traces were included up to approximately $t \approx 400$ min, after which data are incomplete.

REFERENCES

[1] S. Klaus, P. Binder, J. Kim, M. Machado, C. Funaya, V. Schaaf, D. Klaschka, A. Kudulyte, M. Cyrklaff, V. Laketa, T. Höfer, J. Guizetti, N. B. Becker, F. Frischknecht, U. S. Schwarz, and M. Ganter, Science Advances **8**, eabj5362 (2022), https://www.science.org/doi/pdf/10.1126/sciadv.abj5362.
