# Supplementary material for: Competitive resource allocation drives asynchronous and rapid nuclear multiplication in the malaria parasite
Source: Nat Commun. 2026 Jul 27;17:7413. doi: 10.1038/s41467-026-75378-x (PMC13408688; doi:10.1038/s41467-026-75378-x)
Supplement: Supplementary file 3 — Transparent Peer Review file [file 41467_2026_75378_MOESM3_ESM.pdf]

nature portfolio

Peer Review File

Competitive resource allocation drives asynchronous and rapid nuclear multiplication in the malaria parasite

Corresponding Author: Dr Nils Becker

This file contains all reviewer reports in order by version, followed by all author rebuttals in order by version.

Version 0:

Reviewer comments:

Reviewer #1

(Remarks to the Author)
The authors develop a methodology that couples modeling to experimental data that makes the manuscript strong.

The manuscript needs the following revisions:

1. The manuscript is mainly a modeling paper, but there is no literature survey of similar modeling approaches. The cell cycle modeling literature is rich and even if there might not be as many papers for the chosen species, there are certainly similar models developed that uses branching process and two-phase cycles. A comparison of the developed models to existing literature needs to be added.

2. A background on computational models to study the malaria parasite needs to be added to put the developed models into modeling context.

3. A justification of how this work is different than the previous work of the authors (such as [13]) is needed as it is not very clear. The manuscript needs a detailed explanation how the models are different than that were used in the previous work [13].

4. Fig 2c is not easy to follow as there is multiple information presented in one graph.

5. A table summarizing the algorithm for the modeling steps would help with the reproducibility of the modeling results.

Reviewer #2

(Remarks to the Author)
Binder et al investigates why nuclear division(mitosis) during Plasmodium falciparum schizogony in host red blood cell is asynchronous, unlike the synchronous nuclear cycles seen in other multinucleate systems such as Drosophila embryos. Using live-cell imaging of PCNA1-GFP and mathematical modelling, the authors propose that asynchronous nuclear replication arises from competition among nuclei for a shared, limiting pool of DNA replication resources (likely PCNA1 or a related cofactor). They tested two other models models and rule out independent nuclear cycles and inheritance-only models, concluding that inter-nuclear coupling must exist. The paper integrates imaging, statistical analysis, and biophysical modelling to argue that asynchronous replication is an adaptive, resource-efficient strategy rather than a lack of coordination. My knowledge and comments are from the cell biology and the interpretation from biology section. I am not expert in mathematical modelling so cannot comment much on that part.
They tested "limiting pool" model that suggest sequentially based on competition for a limiting pool of a replication resource based on their observation of the sequential localisation pattern of PCNA1::GFP to the nucleus. The authors have conducted a detailed verification of the model based on actual in vivo observation results and have succeeded in deriving a reasonable model, and they have reached a reasonable conclusion that the sequential nuclear multiplication by the model

has an advantage in that it enables more rapid proliferation compared to parallel nuclear multiplication. Sequential allocation of replication resources (as opposed to parallel replication) maximises replication efficiency under resource constraints – effectively allowing P. falciparum to proliferate faster with limited machinery. Simulations reproduce observed patterns of asynchronous replication and predict that this mechanism accelerates schizogony.
While technically solid and well-presented, the work feels a bit incremental relative to existing knowledge from Ganter's own Science Advances paper (Klaus et al., 2022), which already introduced PCNA1-GFP tracking and nuclear desynchrony. This manuscript largely reframes those observations with a theoretical model rather than introducing substantial new biology or molecular mechanism or using another marker like Orc,CDT1 or centrin-Cherry along with PCNA-GFP
It seems that the paper would be further improved if a few more in vivo observations were added, such as whether a similar localisation pattern can be seen with DNA replication markers other than PCNA1::GFP, and whether DNA replication is truly occurring in concert with the localisation of PCNA1::GFP.
Some specific points
1. As mentioned above, the authors discuss and conclude the model for the entire paper by only looking at the localisation pattern of PCNA1::GFP in schizogony, but can a similar sequential nuclear localisation pattern be observed with other DNA replication markers during schizogony?
2. In the time-lapse observation of PCNA1::GFP, has it already been confirmed that the DNA content within the nucleus also increases sequentially, consistent with the sequential localisation pattern of PCNA1::GFP? Would it be possible to confirm this by performing live-cell imaging in a medium containing something like Hoechst?
3. The label for "Fig. 2c, left panel" in Line 138 is incorrect (is Fig. 2d correct?).
4. This may be beyond the scope of the paper, but although the authors have derived a model for non-resource limited cells in addition to the resource-limited condition, what would the DNA replication pattern be when PCNA1::GFP or other DNA replication-related proteins are overexpressed? Would the pattern fit your model?
5. Has the phenomenon that DNA replication proteins like PCNA1 are reused in different nuclei by shuttling between the nucleoplasm and cytoplasm already been proven or directly observed in previous studied? This may also be beyond the scope of the paper, but from the results of your time-lapse observation of PCNA1::GFP, is it possible to see a localisation pattern where PCNA1::GFP moves from the nucleus to the cytoplasm and then localises to another nucleus again?
The central "resource-limitation" concept, while elegant, is a biophysical abstraction lacking direct experimental validation (e.g. identification or manipulation of the proposed limiting factor).

Reviewer #3

(Remarks to the Author)
I co-reviewed this manuscript with one of the reviewers who provided the listed reports. This is part of the Nature Communications initiative to facilitate training in peer review and to provide appropriate recognition for Early Career Researchers who co-review manuscripts.

Reviewer #4

(Remarks to the Author)
This study combines live-cell imaging and mechanistic modeling to explain why Plasmodium falciparum nuclei replicate asynchronously despite sharing a common cytoplasm. By tracking PCNA1::GFP dynamics, the authors show that simultaneous entry of sister nuclei into S-phase slows replication, consistent with competition for a shared cytoplasmic pool of replication factors.

The stochastic branching model with a limiting shared resource reproduces both the bimodal S-phase delay distribution and the deceleration of nuclear cycles during schizogony. Incorporating a late-stage saturation of the resource pool captures the observed re-synchronization near segmentation. Together, the work offers a plausible kinetic framework linking asynchronous replication to efficient parasite proliferation.

Overall, the manuscripts presents an elegant integration of quantitative modeling and microscopy that yields a coherent mechanistic hypothesis for asynchronous nuclear replication. The mathematical formulation is sound, and the qualitative correspondence to data is convincing. However, the manuscript reads primarily as a quantitative biophysical analysis rather than a general mechanistic biology paper. The extensive mathematical detail (e.g., kinetic ODEs and parameter tables) could overwhelm a broad audience. I suggest streamlining the main text for conceptual clarity and wider accessibility, moving derivations to the Supplementary Information, and emphasizing biological implications over the modeling itself.

Major Comments

1. Validation of the limiting-factor hypothesis

The model's central assumption—that DNA replication is limited by a shared factor such as PCNA—is not directly tested. PCNA1::GFP dynamics are consistent with this idea but remain correlative. Inducible PCNA1 overexpression or knockdown could directly test whether S-phase duration and delay distributions depend on PCNA abundance. The model predicts that PCNA overexpression would reduce the coupling between sister nuclei, whereas depletion would accentuate these effects.

2. Parameter identifiability and uncertainty

Model calibration is qualitative—parameters are tuned to reproduce distributions without confidence intervals or sensitivity analysis. Including sensitivity analysis, particularly for (k_u), (k_b), and (r(t)), would strengthen rigor and interpretability.

3. Intermediate kinetic regimes

The manuscript contrasts two extremes—parallel versus sequential replication. Exploring intermediate (k_u) values would clarify how synchronization and S-phase coupling evolve continuously across regimes and identify quantitative thresholds between them.

4. Empirical resource dynamics

The late-stage synchronization is modeled by substituting a measured PCNA1::GFP curve for (r(t)), effectively forcing a saturating resource pool. This is reasonable, but the authors should clarify this substitution explicitly and discuss its biological interpretation (e.g., transcriptional upregulation, increased import, or saturation effects).

5. Accessibility and presentation

Mathematical derivations and parameter-heavy sections could be shifted to the Supplement, replaced in the main text by intuitive schematics of resource competition models. This would make the study more accessible to a broad biological readership.

Minor Comments

1. Define resource explicitly in the Introduction and clarify whether PCNA is the most plausible molecular candidate.
2. Add a schematic early in the Results contrasting Models 1–3 to orient readers.
3. Include a parameter table listing each symbol, biological meaning, units, and estimation method.
4. Clarify how PCNA signal intensity is tracked and normalized to define (r(t)). Tracking details are only summarized. Briefly restating the image analysis workflow—segmentation of nuclei, extraction of nuclear PCNA1::GFP intensity, and thresholding for S-phase detection—would make the manuscript more self-contained.
5. Improve figure color schemes (e.g. Fig. 2D shading)..
6. Clarify numbering of nuclear generations in captions.

Version 1:

Reviewer comments:

Reviewer #2

(Remarks to the Author)
The authors have clarified lot of my comments and their new experiments and also clearly defining how this data different from their previous paper of 2022 makes it very helpful. It has improved very much from previous version and no more comments from us.

(Remarks on code availability)

Reviewer #3

(Remarks to the Author)
I co-reviewed this manuscript with one of the reviewers who provided the listed reports. This is part of the Nature Communications initiative to facilitate training in peer review and to provide appropriate recognition for Early Career Researchers who co-review manuscripts.

(Remarks on code availability)
The code is a usable resource for the community and provided with enough instructions.

Reviewer #4

(Remarks to the Author)
The authors have revised the manuscript and have addressed my comments. Thank you.

(Remarks on code availability)

Open Access This Peer Review File is licensed under a Creative Commons Attribution 4.0 International License, which permits use, sharing, adaptation, distribution and reproduction in any medium or format, as long as you give appropriate credit to the original author(s) and the source, provide a link to the Creative Commons license, and indicate if changes were made.
In cases where reviewers are anonymous, credit should be given to 'Anonymous Referee' and the source.
The images or other third party material in this Peer Review File are included in the article's Creative Commons license, unless indicated otherwise in a credit line to the material. If material is not included in the article's Creative Commons license and your intended use is not permitted by statutory regulation or exceeds the permitted use, you will need to obtain permission directly from the copyright holder.
To view a copy of this license, visit https://creativecommons.org/licenses/by/4.0/

We are very grateful for the time and effort invested by the four reviewers into the assessment of our manuscript. We would like to thank them for the comments and constructive criticism, which were instrumental to significantly improve the quality of our now revised manuscript, as detailed further below.

In our revised manuscript, we have made our work more accessible to the broad audience of ***Nature Communications***, by explaining our mathematical model better in the main text, and by moving more technical aspects into the supplement. The model is now fitted using a rigorous ABC-procedure. We also present new experimental data that support our model. While modulations of PCNA1-expression were suggesting (in accordance with theoretical expectation) that this single factor is not rate-limiting when perturbed in isolation, we were able to exploit a previously unrecognized feature in the measured lineage trees. Here, stratifying our nuclear lineages depending on the S-phase speed of the mother nucleus turned out to be very informative. We believe that our work sheds new light on how ***P. falciparum*** can proliferate so efficiently, which was possible only by tight integration of mathematical modelling and imaging at the limit what is currently possible in ***P. falciparum***. With this interdisciplinary approach, our work fits very well to the scope of ***Nature Communications*** and we are hopeful that the revised version can be accepted for publication.

Reviewer #1 (Remarks to the Author):

The authors develop a methodology that couples modeling to experimental data that makes the manuscript strong.

The manuscript needs the following revisions:

1. The manuscript is mainly a modeling paper, but there is no literature survey of similar modeling approaches. The cell cycle modeling literature is rich and even if there might not be as many papers for the chosen species, there are certainly similar models developed that uses branching process and two-phase cycles. A comparison of the developed models to existing literature needs to be added.

We agree that there is a rich literature on models of cell cycle progression or cell cycle control, including the use of branching processes, and that they are an important background for our own work. We now included a statement mentioning and acknowledging this work at the end of the introductory paragraphs.

2. A background on computational models to study the malaria parasite needs to be added to put the developed models into modeling context.

We agree that mathematical models are an essential element of research on malaria. We have added references to models used to predict the number of cases in a changing environment (e.g., PMID: 41606340) and the rate of proliferation in patients (PMID: 37336700) in the Introduction. To the best of our knowledge, apart from our own earlier work (PMID: 35353560, Ref. [13]), there are no mathematical models concerning nuclear multiplication in ***Plasmodium***.

3. A justification of how this work is different than the previous work of the authors (such as [13]) is needed as it is not very clear. The manuscript needs a detailed explanation how the models are different than that were used in the previous work [13].

We thank the reviewer for pointing out that we insufficiently explained our motivation for this work and how it is different from previous theoretical and experimental results. In brief, in Klaus et al., 2022 we showed that asynchronous nuclear cycles occur (not only asynchronous nuclear division but also DNA replication) and that asynchronous sister nuclei are faster than synchronous ones. In the current

manuscript, we study a mechanism that explains how asynchrony can be established and provide evidence why it is faster.

Due to the limits of light microscopic resolution, nuclear cycles in ***P. falciparum*** cells with many dynamically dividing nuclei cannot be investigated experimentally. Thus, in Klaus et al., 2022 (PMID: 35353560), we developed a mathematical model with independent nuclear phases, essentially allowing us to extrapolate the experimentally determined dynamics of early cycles (few nuclei per cell) to late cycles (many nuclei per cell). This led to the discovery that the slow synchronous sister phases delay nuclear multiplication as a whole and, hence, we concluded that asynchrony facilitates rapid proliferation.

In this manuscript, we asked how asynchrony of nuclear cycles can arise mechanistically. To this end, we started with minimal mathematical models, aiming to reproduce our experimental data (with new experimental data added to the one already used in Klaus et al., 2022, PMID: 35353560). Since models without a physical coupling of the nuclei proved insufficient, we developed a new model based on a biophysically plausible coupling mechanism (resource sharing), which recapitulated all key features of our experimental data. This model, for the first time, provides an explanation how asynchronous nuclear cycles arise and why they are faster than synchronous ones.

Throughout the revised manuscript, we now clearly separate our previous work from this manuscript. For example, in the subsection entitled “A limiting pool mechanism couples replicating nuclei”, before introducing the current model (“model 3”), we now refer explicitly to the model in Klaus et al., 2022 and outline the difference.

4. Fig 2c is not easy to follow as there is multiple information presented in one graph.

We agree that the figure was very dense. In the resubmitted manuscript, we split the panel (now 2d) into two separate parts for input to the model and output from the model, which should help readability.

5. A table summarizing the algorithm for the modeling steps would help with the reproducibility of the modeling results.

We agree that reproducibility is essential. We have made an effort to provide full detail on the definition and simulation of the three models as part of the Supplementary Methods, which then is referenced in the main text. In particular, we have added a section on model 1 for completeness, and reviewed the sections on model 2 for completeness and accessibility. Model 3 gained a symbol overview table, and a critical review of the supplementary text. We also hope that the new placement of model derivations in the Method sections helps with readability, as all mathematical details necessary for reproducing are collected together.

Reviewer #2 (Remarks to the Author):

Binder et al investigates why nuclear division(mitosis) during Plasmodium falciparum schizogony in host red blood cell is asynchronous, unlike the synchronous nuclear cycles seen in other multinucleate systems such as Drosophila embryos. Using live-cell imaging of PCNA1-GFP and mathematical modelling, the authors propose that asynchronous nuclear replication arises from competition among nuclei for a shared, limiting pool of DNA replication resources (likely PCNA1 or a related cofactor). They tested two other models and rule out independent nuclear cycles and inheritance-only models, concluding that inter-nuclear coupling must exist. The paper integrates imaging, statistical analysis, and

biophysical modelling to argue that asynchronous replication is an adaptive, resource-efficient strategy rather than a lack of coordination.
My knowledge and comments are from the cell biology and the interpretation from biology section. I am not expert in mathematical modelling so cannot comment much on that part.
They tested "limiting pool" model that suggest sequentially based on competition for a limiting pool of a replication resource based on their observation of the sequential localization pattern of PCNA1::GFP to the nucleus. The authors have conducted a detailed verification of the model based on actual in vivo observation results and have succeeded in deriving a reasonable model, and they have reached a reasonable conclusion that the sequential nuclear multiplication by the model has an advantage in that it enables more rapid proliferation compared to parallel nuclear multiplication. Sequential allocation of replication resources (as opposed to parallel replication) maximises replication efficiency under resource constraints – effectively allowing P. falciparum to proliferate faster with limited machinery. Simulations reproduce observed patterns of asynchronous replication and predict that this mechanism accelerates schizogony.
While technically solid and well-presented, the work feels a bit incremental relative to existing knowledge from Ganter's own Science Advances paper (Klaus et al., 2022), which already introduced PCNA1-GFP tracking and nuclear desynchrony. This manuscript largely reframes those observations with a theoretical model rather than introducing substantial new biology or molecular mechanism or using another marker like Orc, CDT1 or centrin-Cherry along with PCNA-GFP.
It seems that the paper would be further improved if a few more in vivo observations were added, such as whether a similar localization pattern can be seen with DNA replication markers other than PCNA1::GFP, and whether DNA replication is truly occurring in concert with the localization of PCNA1::GFP.

We apologize for not clearly separating our current work from our previous publication. Klaus et al., 2022 showed that asynchrony exists (i.e., not only asynchronous nuclear division but also DNA replications) and that asynchronous first-generation sisters replicate faster than synchronous ones. Here, we develop a novel theoretical and mechanistic description of the underlying mechanisms. It shows how asynchrony can be established (by a limiting replication resource that can be bound to DNA) and why it is faster (because resource idling times are minimized). Importantly, we also test the new model with new data from a different experimental setup with different growth conditions compared to Klaus et al, 2022. Throughout the revised manuscript, we now clearly separate that previous work from this manuscript. For example, in the subsection entitled "A limiting pool mechanism couples replicating nuclei", before introducing the current model ("model 3"), we now refer explicitly to the model in Klaus et al., 2022 and outline the difference. In addition, we did test different replication markers, however, these experiments were not rewarding as detailed below.

Some specific points
1. As mentioned above, the authors discuss and conclude the model for the entire paper by only looking at the localization pattern of PCNA1::GFP in schizogony, but can a similar sequential nuclear localization pattern be observed with other DNA replication markers during schizogony?

We tried to identify additional replication markers similar to PCNA1, however, thus far, unsuccessful. For example, we GFP-tagged replication protein A, which is thought to stabilize single stranded DNA at the replication forks, and we tagged two subunits of replication factor C (RFC), which in other organisms loads the PCNA homotrimer onto the DNA. However, all three candidates localized pan-cellularly and did not accumulate in S-phase nuclei. Similarly, ectopic expression of DNA polymerase delta fused to GFP resulted in a pan-cellular signal, which was stable over time. Preliminary results from a dual reporter line, expressing PCNA1::GFP and PCNA2::mCherry indicate that PCNA2 does not behave like PCNA1. We and others have shown that centrins localize persistently to the centriolar

plaque (e.g., PMIDs: 38150475, 30541825). Thus, we currently only know of PCNA1 showing such a dynamic localization pattern. CDT1 is certainly an interesting protein, which is likely involved in origin licensing and we currently fucus on proteins involved in origin of replication firing as prime candidates, but these await experimental characterization.

2. In the time-lapse observation of PCNA1::GFP, has it already been confirmed that the DNA content within the nucleus also increases sequentially, consistent with the sequential localization pattern of PCNA1::GFP? Would it be possible to confirm this by performing live-cell imaging in a medium containing something like Hoechst?

Indeed, we had already reported the results of the suggested experiment in Klaus et al., 2022 (PMID: 35353560), which showed a doubling of the nuclear DNA content specifically during the time interval where PCNA1 is accumulated in a nucleus. We also used the PCNA1::GFP localization to assess the effect of drugs interfering with DNA replication (e.g., aphidicolin). Coherently, we found that perturbing DNA replication affects PCNA1::GFP localization (PMIDs: 37345936, 41042506), which we now clearly mention in the revised manuscript.

Thus, we are confident that PCNA1::GFP is a robust marker for DNA replication, regardless of whether PCNA1 is or is not part of the limiting factor R that we show to control replication speed.

3. The label for “Fig. 2c, left panel” in Line 138 is incorrect (is Fig. 2d correct?).

Thanks for pointing out this mistake; it should be Fig. 2d indeed. This has been corrected.

4. This may be beyond the scope of the paper, but although the authors have derived a model for non-resource limited cells in addition to the resource-limited condition, what would the DNA replication pattern be when PCNA1::GFP or other DNA replication-related proteins are overexpressed? Would the pattern fit your model?

We thank the reviewer for this insightful comment. Indeed, we were able to provide additional experimental support, further validating our model.

Beforehand, we would like to remark that overexpression of a single factor may not be sufficient to relieve resource limitation, based on a theoretical argument. Systems limited by general scarcity of available building blocks operate (Pareto-)efficiently only if none of the components is in excess relative to the others. These effects are well known for metabolic pathways: Metabolic control analysis (reviewed in PMID: 18629230) shows that overexpression of a single enzyme of pathway does not translate into more flux, and a substantial increase is seen only when the abundance of multiple components is modulated simultaneously.

Our model 3 predicts that conditions of higher resource availability should yield a clear phenotype, including much slower desynchronization. We believe that our second set of tracking experiments conducted on the Zeiss Airyscan microscope do correspond to such conditions, because phototoxic effects slow down cycle progression significantly (see SI Fig. S7 in the supplementary information, previously S5), allowing resource production to keep up better effectively making it more abundant. As we show, these experiments exhibit the phenotype expected for higher abundance, and model 3 was able to reproduce it with a single parameter change.

In addition, and motivated by this and similar comments from the other reviewers, we have now conducted the suggested direct perturbation experiments. Unfortunately, we could neither detect a

phenotype for overexpression nor for depletion of PCNA1. Please see below (response to reviewer 4, major comment 1) for a detailed description of these experiments, the additional data (presented for the reviewers in Fig.1), and an interpretation of these results.

Nonetheless, we were able to provide new experimental evidence by an alternative route, independent of PCNA1 perturbation. We exploited the natural variation in S-phase duration in mother nuclei and its correlation to the respective daughter nuclei. We hypothesized that if our limiting-factor hypothesis is valid, rapid DNA replication in the mother nucleus is enabled by an above-average amount of resource R, rather than, e.g., reduced transcriptional activity or increased nucleotide abundance. Consequently, daughter nuclei of very rapidly replicating mother nuclei (i.e., in parasites where resource is abundant) should be less affected by resource sharing. In particular,

(i) when replicating synchronously, their S-phases should maintain near full speed, and
(ii) when replicating asynchronously, queueing for resource should end quicker and we expect more events with a replication delay between 0 and 1, i.e., overlapping S-phases.

Indeed, when we stratified our experimental data depending on the S-phase speed of the mother nucleus, these predictions were fully confirmed.

As an alternative explanation, one could consider the variability in mother DNA replication to be caused by varying nucleotide abundance. However, this mechanism cannot explain the observed differential effect on the replication speed in synchronous vs. asynchronous daughter S-phases, the shift in the distribution of daughter replication delays, and the fact that nuclei replicate at different speeds at all.

These new data and analysis are shown in the new panels Fig. 6cde, lending further experimental support to our main conclusion that competitive allocation of a resource drives asynchronous nuclear cycles during ***P. falciparum*** blood stage schizogony.

For further clarification and to avoid unintentionally suggesting that PCNA1 may be the single limiting resource, we removed all mentions of PCNA1::GFP in the results section, except of in the context of the nuclear cycle sensor line. As mentioned below, whether PCNA1 can become limiting (or not) needs additional experimental investigation. We also added a short discussion of the potential role of PCNA1 in the Discussion section.

5. Has the phenomenon that DNA replication proteins like PCNA1 are reused in different nuclei by shuttling between the nucleoplasm and cytoplasm already been proven or directly observed in previous studies?
This may also be beyond the scope of the paper, but from the results of your time-lapse observation of PCNA1::GFP, is it possible to see a localisation pattern where PCNA1::GFP moves from the nucleus to the cytoplasm and then localises to another nucleus again?
The central “resource-limitation” concept, while elegant, is a biophysical abstraction lacking direct experimental validation (e.g. identification or manipulation of the proposed limiting factor).

We are not aware of literature directly showing that individual ***P. falciparum*** DNA replication proteins are reused in different nuclei by shuttling between the nucleoplasm and cytoplasm. However, in the context of origin of replication firing within a nucleus, the reuse of DNA replication proteins has been previously described, and, in fact, origins compete for multiple proteins involved in origin firing, e.g., Sld2, 3, and 7 (PMID: 22081107; PMID: 22169533).

Our own data published in Klaus et al., 2022, Fig. 2c (PMID: 35353560), do show that the cytoplasmic signal of PCNA1 decreases as the nuclear signal increases at the onset of S-phase, and the reverse as S-phase concludes, while the total fluorescence remains nearly constant throughout. This strongly

suggests that all nuclei access the same pool of protein and that PCNA1 shuttles between nuclei. We aim to test this directly with PCNA1 fused to a photoconvertible fluorophore, but a direct proof was not possible yet.

In the revised discussion, we now included a section where we describe the competition among origins of replication and that these proteins appear as good candidates for components of the resource.

Reviewer #3 (Remarks to the Author):

I co-reviewed this manuscript with one of the reviewers who provided the listed reports. This is part of the ***Nature Communications*** initiative to facilitate training in peer review and to provide appropriate recognition for Early Career Researchers who co-review manuscripts.

We very much appreciate the involvement of early career researchers in the revision process and are grateful for the constructive comments.

Reviewer #4 (Remarks to the Author):

This study combines live-cell imaging and mechanistic modeling to explain why Plasmodium falciparum nuclei replicate asynchronously despite sharing a common cytoplasm. By tracking PCNA1::GFP dynamics, the authors show that simultaneous entry of sister nuclei into S-phase slows replication, consistent with competition for a shared cytoplasmic pool of replication factors.

The stochastic branching model with a limiting shared resource reproduces both the bimodal S-phase delay distribution and the deceleration of nuclear cycles during schizogony. Incorporating a late-stage saturation of the resource pool captures the observed re-synchronization near segmentation. Together, the work offers a plausible kinetic framework linking asynchronous replication to efficient parasite proliferation.

Overall, the manuscript presents an elegant integration of quantitative modeling and microscopy that yields a coherent mechanistic hypothesis for asynchronous nuclear replication. The mathematical formulation is sound, and the qualitative correspondence to data is convincing. However, the manuscript reads primarily as a quantitative biophysical analysis rather than a general mechanistic biology paper. The extensive mathematical detail (e.g., kinetic ODEs and parameter tables) could overwhelm a broad audience. I suggest streamlining the main text for conceptual clarity and wider accessibility, moving derivations to the Supplementary Information, and emphasizing biological implications over the modeling itself.

We agree. The manuscript was initially written with a strong focus on the mathematical model, but for the general audience of ***Nature Communications***, the presentation should be more accessible. We have thoroughly revised the manuscript for greater accessibility to a broader audience, by transferring mathematical derivations and model details to the Methods section. The main text was also streamlined, keeping only the definitions of central quantities that are essential to keep the presentation self-contained. We also simplified the notation in several places.

Major Comments

1. Validation of the limiting-factor hypothesis

The model's central assumption—that DNA replication is limited by a shared factor such as PCNA—is not directly tested. PCNA1::GFP dynamics are consistent with this idea but remain correlative. Inducible PCNA1 overexpression or knockdown could directly test whether S-phase duration and delay distributions depend on PCNA abundance. The model predicts that PCNA1 overexpression would reduce the coupling between sister nuclei, whereas depletion would accentuate these effects.

We thank the reviewer for this insightful suggestion, which addresses the core of our argument.

Please note that we do not intend to claim in this work that PCNA1 is the shared factor, although our model is motivated by our data on PCNA1. Our main contribution is a biophysically consistent mechanism for nuclear cycle dynamics that explains the data in a consistent fashion (even if the molecular players are not identified).

We fully agree with the reviewer that direct perturbations would be highly informative. Thus, taking up the point from this and another reviewer, we have been working towards challenging the model with additional experimental data. Our new findings are detailed below. In brief, an effect of different endogenous PCNA1 levels on S-phase dynamics could not be assessed. Also, overexpression and inducible knockdown of PCNA1 were insufficient to exhibit a phenotype, not permitting a direct test of the predictions of our model (presented for the reviewer in Fig. 1 below). For further clarification and to not indirectly imply that PCNA1 may be the limiting resource, we thus removed all mentions of PCNA1::GFP, except of in the context of the nuclear cycle sensor line. Whether PCNA1 can become limiting (or not) needs additional experimental investigation, which might become more feasible in the future with advances in ***P. falciparum*** manipulation and imaging technology.

Nonetheless, we were able to add an alternative new analysis based on stratifying data based on mother S-phase duration, through which we provide additional experimental support for resource sharing independent of a PCNA1 perturbation.

Correlation of endogenous PCNA1 levels with S-phase dynamics:
To test the effect of different expression levels on S-phase dynamics and nuclear coupling, we tried to determine the endogenous PCNA1 expression levels. However, a viable cell line with a C-terminal T2A skip peptide followed by GFP, in which the GFP fluorescence would be directly proportional to endogenous PCNA1 abundance, was never obtained. This indicates that the 8 amino acid modification from the skip peptide was not tolerated. Indeed, on the ***Plasmodium*** data base PlasmoDB, no non-synonymous SNPs have been described for PCNA1, supporting the notion that any protein modification is likely detrimental.

Overexpression of PCNA1:
This problem could be circumvented by overexpressing PCNA1, and we did (see further below). However, a theoretical argument can be made that this is unlikely to work. Systems limited by general scarcity of available building blocks operate (Pareto-)efficiently only if none of the components is in excess relative to the others. These effects are well known for metabolic pathways: Overexpression of a single enzyme of pathway does not translate into more flux and a substantial increase is seen only when the abundance of multiple components is modulated simultaneously. This is one of the major conclusions of metabolic control analysis (e.g., reviewed in PMID: 18629230).

In a similar fashion, in budding yeast, origins-of-replication compete for multiple proteins involved in origin firing (e.g., Sld2, 3, and 7; PMID: 22081107; PMID: 22169533), making the latter collectively limiting. It is plausible that ***P. falciparum*** is efficient in the same sense, so that overexpression of PCNA1

would likely make one or more other components limiting, not achieving a net reduction in resource limitation, which would make that experiment inconclusive.

REDACTED

While somewhat disappointing, these results are in line with previous published work from the Höfer group (PMID: 24499930). In a study of nucleotide-excision repair in a mammalian cell line, a two-fold lower or higher concentration of PCNA did not affect the rate of DNA repair, and this robustness was also observed for other individual components of the repair machinery. Instead, the rate of repair is controlled collectively by all components, as an effect of the multistep assembly of the repair machinery, which is slow compared to the individual components' kinetics. Extending the optimality argument given above, this finding indicates that the abundance of any individual component protein in a knockdown would need to fall below a very low threshold to reveal a phenotype. For PCNA1, the glmS-based inducible knockdown appears to be insufficient. Due to collective control, the unidentified resource R in our present work may be best envisioned as a set of several diffusible replication cofactors that together activate transcription forks. We have updated the section introducing model 3 to reflect this possibility.

Experimental support independent of PCNA1 perturbation:

As PCNA1 perturbation was not effective and, indeed, the theoretical considerations above suggest that it is very unlikely to elicit a strong phenotype by varying levels of a single protein (note that this is qualitatively different from complete knockouts, which of course generally will have phenotypes), the question arises how the level of resource abundance can be perturbed experimentally. In the original manuscript, we had already reported on a repeat experiment, which was conducted under conditions of higher phototoxicity on the Zeiss Airyscan microscope. There, nuclear cycles were generally slowed down, and nuclear synchrony was increased noticeably. These observations can be explained by a single parameter change in model 3, which puts it in a regime of higher resource abundance. Although indirect, we believe this remarkable agreement provides additional experimental support for the limiting

factor hypothesis. We have reworked the corresponding text at the end of the Results section to make this point more clearly.

In addition, this observation indicated that global differences can be informative and we harnessed the natural variation in S-phase duration in mother nuclei to further test our model.

Additional experimental support independent of PCNA1 perturbation:
As an alternative route, we exploited the natural variation in S-phase duration in mother nuclei as well as its correlation to the respective daughter nuclei. We hypothesized that if the limiting-factor hypothesis is valid, rapid DNA replication in the mother nucleus is enabled by an above-average amount of resource (rather than, e.g., different transcriptional activity or increased nucleotide abundance). Consequently, daughter nuclei of very rapidly replicating mother nuclei, i.e., in parasites where resource is abundant, should be less affected by resource sharing. In particular,

(i) when replicating synchronously, their S-phases should maintain near full speed, and
(ii) when replicating asynchronously, queueing for resource should end quicker and we expect more events with a replication delay between 0 and 1, i.e., overlapping S-phases.

Indeed, these predictions were confirmed by stratifying our experimental data depending on the S-phase speed of the mother nucleus.

As an alternative explanation, one could consider the variability in mother DNA replication to be caused by varying nucleotide abundance. However, this mechanism cannot explain the observed differential effect on the replication speed in synchronous vs. asynchronous daughter S-phases, the shift in the distribution of daughter replication delays, and the fact that nuclei replicate at different speeds at all.

These new data and analysis are shown in the new panels of Fig. 6cde, lending further experimental support to our main conclusion that competitive allocation of a resource drives asynchronous nuclear cycles during ***P. falciparum*** blood stage schizogony.

2. Parameter identifiability and uncertainty

Model calibration is qualitative—parameters are tuned to reproduce distributions without confidence intervals or sensitivity analysis. Including sensitivity analysis, particularly for (k_u), (k_b), and (r(t)), would strengthen rigor and interpretability.

We thank the reviewer for suggesting this improvement. We have now carried out a thorough and systematic model inference using Approximate Bayesian Computing, based on forward simulations of the resource model 3. As distance metric in ABC, we used the deviation of the predicted vs. observed distributions of 2-nuclei stage sister S-phase lengths. We found that our data constrain the parameters rather well. In particular, k_u is inferred to have an upper bound, clearly indicating that sequential-mode replication is required to fit the data. Moreover, k_b is well determined, which probably stems from the fact that together with the D-phase variability σ_D, it controls the fraction of simultaneous sister nuclei. We also inferred the support range for ξ, which sets the overall resource availability and its variability. This showed that resource variability among cells is required to reproduce the observed S-phase variability. Although the inference used only the two-nuclei stage, the MAP parameters also fitted the longer-term data on desynchronization over and total duration of schizogony surprisingly well. The extrapolation relies on a saturating resource level as a function of time r(t), as in the initial submission. For the new fit procedure, r(t) was improved by better respecting the normalization inherent in the data, and including all our data from both PCNA1::GFP and NLS::mCherry, which led to slight changes in shape.

The new inference procedure is described in Methods. The results are presented in the updated Fig. 6 in the main text. Details are explained, and full posterior plots are given in Supplementary Fig. 5.

In Fig. 6f, the desynchronization profile obtained with the new inferred parameters is compared to experimental data. We discovered a slight inconsistency in the original experimental profile, in that traces that did not cover the full duration of schizogony until egress were included in Fig. 6f, but the box plots of the total time to the last S-phase (Fig. 6g) did not include those traces. To fix this, we excluded traces not covering the full duration from Fig. 6f. This led to a very slight alteration of the profile in Fig. 6f, which did not change any conclusions.

3. Intermediate kinetic regimes

The manuscript contrasts two extremes—parallel versus sequential replication. Exploring intermediate (k_u) values would clarify how synchronization and S-phase coupling evolve continuously across regimes and identify quantitative thresholds between them.

Thank you for this interesting comment. As described above, our new fit shows that the data support only the sequential regime, and we find using the slower parallel mode as a point of comparison to be the most illustrative.

We agree it is desirable to give a complete picture of the model's behavior. To this end, we have now included in Supplementary Fig. 3 simulation results in analogy to Fig. 5 of the main text, for the intermediate regime where k_u is comparable to the S-phase length. Moreover, Supplementary Fig 3c shows the growth advantage as a function of the unbinding rate, mapping the full range of synchronization dynamics in the model. We now mention the intermediate regime when introducing and discussing model 3 and refer to the intermediate dynamics in the context of Fig. 5.

4. Empirical resource dynamics

The late-stage synchronization is modeled by substituting a measured PCNA1::GFP curve for (r(t)), effectively forcing a saturating resource pool. This is reasonable, but the authors should clarify this substitution explicitly and discuss its biological interpretation (e.g., transcriptional upregulation, increased import, or saturation effects).

Thank you for pointing out our lack of clarity. We agree that this point deserves more attention. Indeed, the saturating growth of the resource, combined with the near-exponential growth in nuclear number implies that resource scarcity tends to become more pronounced towards the end of schizogony. This leads to an overall gradual slowdown, in agreement with the (then unexplained) observation we made already in Klaus et al, 2022 (PMID: 35353560).

Although our available temporal profiles are best for PCNA1::GFP, also the 3xNLS::mCherry traces show a similar saturating behavior over time. Several other studies indicate a general reduction in protein translational activity in the second half of schizogony, e.g.; PMIDs: 25493618. 27230797, 29985403.

As part of our improved fitting procedure, we have used an average of PCNA1::GFP and 3xNLS::mCherry time courses to determine r(t), now shown in Supplementary Fig. 4. In the revised manuscript, we also devote more explanatory text to the resource model including its biological

interpretation, in the subsection entitled "A limiting pool mechanism can explain asynchronous nuclear multiplication in ***P. falciparum***".

5. Accessibility and presentation

Mathematical derivations and parameter-heavy sections could be shifted to the Supplement, replaced in the main text by intuitive schematics of resource competition models. This would make the study more accessible to a broad biological readership.

We appreciate this comment. In the revised version, we strive to address the broad audience of ***Nature Communications*** better, by moving derivations to Methods, and further mathematical and inference details to the Supplement. In the process, the main text was thoroughly reworked becoming, as we hope, more readable. Because some of the main results are theoretical, we had to keep the essential mathematical symbols and basic equations. The main model 3 is schematized in Fig. 3.

Minor Comments

1. Define resource explicitly in the Introduction and clarify whether PCNA1 is the most plausible molecular candidate.

This is an important point, which we insufficiently addressed for the initial submission. At the end of the introduction and in the subsection “A limiting pool mechanism couples replicating nuclei”, we now clearly state that the resource is likely one or multiple proteins which control the speed of DNA replication by their (collective) activity, and can translocate between nuclei. We also discuss PCNA1 specifically as a plausible component of the resource in the Discussion.

2. Add a schematic early in the Results contrasting Models 1–3 to orient readers.

We agree on the value of comparative schematics. As we test the model one by one and only introduce model 3 after 1 and 2 fail, we think it suits the reading flow best to not be reminded about models 1 and 2 when discussing model 3. Thus, we would like to respectfully refrain from adding a schematic early on, in addition to the existing schematics for model 3. To orient readers better within the quantities related to model 3, we have also included a symbol table in Methods.

3. Include a parameter table listing each symbol, biological meaning, units, and estimation method.

We have included a legend table for the basic symbols of model 3 in Methods. As part of the new inference, we have also included a parameter results table in Methods.

4. Clarify how PCNA signal intensity is tracked and normalized to define (r(t)). Tracking details are only summarized. Briefly restating the image analysis workflow—segmentation of nuclei, extraction of nuclear PCNA1::GFP intensity, and thresholding for S-phase detection—would make the manuscript more self-contained.

Thanks for this improvement suggestion. Briefly, for tracking, we made use of three transgenic ***P. falciparum*** cell lines, which express fluorescently tagged proteins either episomally (PCNA1::GFP, 3xNLS::mCherry; PMID: 35353560) or endogenously (LRR; see also Fig.1, ***unpublished***). While expression of the latter is controlled by its endogenous promoter, PCNA1::GFP expression is controlled

by the ***P. falciparum*** CRT promoter and 3xNLS::mCherry by the ***P. falciparum*** HSP86 promoter. The commonly used ***P. berghei*** DHFR/TS 3'UTR serves as terminator in all three constructs. To reduce phototoxicity, we select cells for long-term imaging, which showed a good fluorescent signal around the onset of the first S-Phase (i.e., the protein of interest is already present in substantial amounts). For all three cell lines, the fluorescence further increased during imaging. As the PCNA1::GFP and 3xNLS::mCherry cell lines produced a large number of time-series suitable for quantitative analysis, they were chosen to exemplify protein increase over time (now shown in new Supplementary Fig. 4).

We have now extended the Methods section by describing in detail the image analysis workflow to inform r(t).

5. Improve figure color schemes (e.g. Fig. 2D shading).

We went through the figures critically to improve readability. Fig. 2c was simplified for clarity and 2d was improved.

6. Clarify numbering of nuclear generations in captions.

We have now clarified the numbering of nuclear generations in the caption of Fig. 2.
